# Targeting the Mouse Ventral Hippocampus in the Intrahippocampal Kainic Acid Model of Temporal Lobe Epilepsy

**DOI:** 10.1523/ENEURO.0158-18.2018

**Published:** 2018-08-08

**Authors:** Zachary Zeidler, Mikaela Brandt-Fontaine, Caara Leintz, Chris Krook-Magnuson, Tay Netoff, Esther Krook-Magnuson

**Affiliations:** 1 Graduate Program in Neuroscience, University of Minnesota, Minneapolis, MN 55455; 2 Department of Biomedical Engineering, University of Minnesota, Minneapolis, MN 55455; 3 Department of Neuroscience, University of Minnesota, Minneapolis, MN 55455

**Keywords:** anhedonia, animal model, anxiety, depression, epilepsy, intrahippocampal kainic acid

## Abstract

Here we describe a novel mouse model of temporal lobe epilepsy (TLE) that moves the site of kainate injection from the rodent dorsal hippocampus (corresponding to the human posterior hippocampus) to the ventral hippocampus (corresponding to the human anterior hippocampus). We compare the phenotypes of this new model—with respect to seizures, cognitive impairment, affective deficits, and histopathology—to the standard dorsal intrahippocampal kainate model. Our results demonstrate that histopathological measures of granule cell dispersion and mossy fiber sprouting maximize near the site of kainate injection. Somewhat surprisingly, both the dorsal and ventral models exhibit similar spatial memory impairments in addition to similar electrographic and behavioral seizure burdens. In contrast, we find a more pronounced affective (anhedonic) phenotype specifically in the ventral model. These results demonstrate that the ventral intrahippocampal kainic acid model recapitulates critical pathologies of the dorsal model while providing a means to further study affective phenotypes such as depression in TLE.

## Visual Abstract

**Figure:**
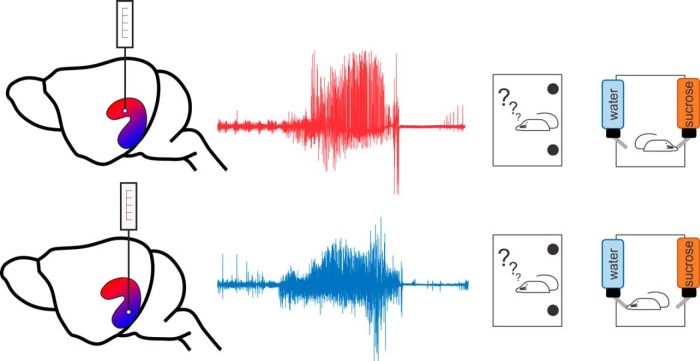

## Significance Statement

Temporal lobe epilepsy (TLE) is characterized by spontaneous recurring seizures. TLE additionally features cognitive and affective comorbidities that impair quality of life. Animal models studying TLE are critical to advance our understanding of the disorder. A current popular model targets the dorsal hippocampus with a focal injection of kainic acid to induce epilepsy. Evidence suggests that targeting the ventral hippocampus may produce a model of TLE with distinct benefits. We present data demonstrating that targeting the mouse ventral hippocampus with kainic acid creates a TLE model with seizure and cognitive phenotypes similar to the standard dorsal model, but with additional, pronounced, affective features, including anhedonia. These results describe a new tool for epilepsy researchers to better study comorbidities of TLE.

## Introduction

Temporal lobe epilepsy (TLE) is the most common form of epilepsy in adults and features spontaneous recurring seizures in addition to cognitive and affective comorbidities, such as depression and anxiety. Despite decades of research, there is still a strong need to better understand various TLE pathologies, as evidenced by the persistence of high rates of comorbid depression (Hermann et al., 2000). Animal models of TLE have been critical in advancing the field’s knowledge of TLE mechanisms and pathologies. One TLE model that has proved to be highly useful is the intrahippocampal kainic acid (IHKA) model. In this model, kainic acid (KA) is injected into the hippocampus, creating a focal insult and an acute, severe ictal period (status epilepticus). Following a latent period, this model produces spontaneous, recurring seizures as well as classic histopathological features also observed in human TLE: sprouting of mossy fibers [mossy fiber sprouting (MFS)], dispersion of granule cells [granule cell dispersion (GCD)], and hippocampal sclerosis (Bouchet and Cazauvieilh, 1825; Andersson et al., 1991; Suzuki et al., 1995; Bouilleret et al., 1999; Arabadzisz et al., 2005). There are multiple advantages to the IHKA model. For one, by localizing the insult to a single region of the hippocampus, KA cannot directly affect other aspects of the CNS or peripheral nervous system (unlike systemic models, including the intraperitoneal KA model). This is important in interpreting extrahippocampal changes following acute insult as being the result of epilepsy-related factors and not a direct effect of the chemoconvulsant. Additionally, the IHKA model has a low mortality rate compared with some other models, such as systemic pilocarpine injection. Overall, the IHKA model is a practical and informative model of TLE that exhibits important phenotypes observed in human TLE.

IHKA was first formally proposed as a model of human TLE decades ago (Cavalheiro et al., 1982; Suzuki et al., 1995; Bouilleret et al., 1999). However, interest in the potential of KA to alter the hippocampal formation, both via systemic and targeted injections, had already been developing by that time (Nadler et al., 1978; Schwarcz et al., 1978). Early focal injections of KA mostly targeted the dorsal hippocampus (Schwarcz et al., 1978; Tanaka et al., 1982), and, despite some interest in investigating how changing the target of KA injection altered the behavioral and histopathological outcomes (Schwob et al., 1980; Cavalheiro et al., 1982), the dorsal hippocampus became the de facto region in which to investigate IHKA and TLE pathology (Suzuki et al., 1995; Bouilleret et al., 1999).

As the epilepsy field cemented the location of IHKA injections to the dorsal hippocampus, hippocampal researchers were bringing to light important distinctions between areas along the septotemporal axis of the hippocampus. For example, the dorsal third of the hippocampus was found to project to the retrosplenial cortex, a region involved in navigation, while the ventral two-thirds of the hippocampus has projections to various amygdalar areas that are involved in emotional response (Cenquizca and Swanson, 2007). While different organizational schemes subdivide the hippocampus into various components (Amaral and Witter, 1989; Fanselow and Dong, 2010; Strange et al., 2014; Shah et al., 2016), one proposal suggests a broad division between the dorsal hippocampus (anterior in rodents, but corresponding to the human posterior hippocampus), which is involved with cognitive and memory functions, and the ventral hippocampus (posterior in rodents, but corresponding to the human anterior hippocampus), which is involved with affective processes (Moser and Moser, 1998).

Distinctions in the dorsal and ventral hippocampus manifest in both healthy and epileptic hippocampi. For example, epileptiform spontaneous bursting was noted to be more frequent in the ventral compared with dorsal hippocampus *in vitro* (Bragdon et al., 1986). Furthermore, seizures evoked by electrical stimulation of the ventral hippocampus generalize with fewer stimulations than evoked dorsal hippocampal seizures (Racine et al., 1977). Importantly, studies in human TLE patients observed that the anterior portion of the human hippocampus (corresponding to the rodent ventral hippocampus) is more ictogenic than the human posterior hippocampus (corresponding to the rodent dorsal hippocampus; Engel et al., 1990).

With mounting evidence of important distinctions between dorsal and ventral aspects of the hippocampus, both in healthy and epileptic tissue, we returned to the IHKA model and asked why it specifically targets the dorsal hippocampus. Functional and connectivity evidence indicates that targeting the ventral hippocampus with a KA injection may create a model of TLE with increased seizures and/or more generalized seizures compared with the dorsal model, a greater disruption of affective processes that could aid in addressing affective comorbidities of human TLE, as well as in establishing greater face validity to the human condition. This line of reasoning led us to target the ventral hippocampus with KA (vKA) and compare epilepsy-related phenotypes to the standard dorsal intrahippocampal KA (dKA) model. Specifically, we sought to address the following. (1) Does the vKA model recapitulate key anatomic pathologies seen in the dKA model? (2) Does the vKA model produce a distinct ictal phenotype from the dKA model, with regard to both electrographic and overt motor seizures? (3) Does the vKA model exhibit cognitive or affective phenotypes? If so, how do these compare to the dKA model?

## Materials and Methods

All animal procedures were performed in accordance with the regulations of the animal care committee of the University of Minnesota.

### Epilepsy induction and electrode implantation

Male (*n* = 24) and female (*n* = 33) C57B/6J mice were stereotaxically injected with either 100 nl of saline or 100 nl of 20 mm KA in saline at postnatal day 45 or greater (mean ± SD: dKA = 60 ± 19 d; vKA = 59 ± 20 d; saline = 69 ± 23 d). Injection coordinates for the dorsal hippocampus group, in centimeters from bregma, were as follows: anteroposterior (AP), −0.2; mediolateral (ML), 0.125; dorsoventral (DV), −0.16. Coordinates for the ventral hippocampus were as follows: AP, −0.36; ML, 0.28; DV, −0.28. After injection of saline or KA, the syringe was immediately withdrawn and the animal was returned to its home cage to recover. We observed no acute mortality with this dose of KA in these animals. One week after saline or KA injection, animals were implanted with a twisted wire bipolar electrode (Plastics One) approximately equidistant between the dorsal and ventral injection sites (AP, 0.28; ML, 0.25; DV, −0.2). During all surgeries, animals were maintained on isoflurane anesthesia (∼2%) and a heating pad. Animals were group housed until the electrode implantation, at which point they were singly housed. No significant differences were observed between dorsally and ventrally injected saline mice; therefore, the saline subjects were collapsed into one group.

### Chronic video and local field potential monitoring and analysis

Approximately 28 d postinjection (mean ± SD: dKA = 27 ± 14 d; vKA = 24 ± 14 d; saline = 20 ± 9 d), animals were chronically housed with simultaneous video and local field potential (vLFP) monitoring (Krook-Magnuson et al., 2013). A subset of animals was additionally rerecorded at a later time point (dKA = 203 ± 11 d post-KA; vKA = 211 ± 13 d post-KA). Electrical patch cables were connected to a commutator and then amplified 5000–10,000 times (Brownlee Precision 410, Neurophase) before recording. Signal was obtained from the local differential of the two twisted wires of the electrode. Data from vLFP monitoring were continuously gathered for 2 weeks using custom Matlab-based software (a previous version of which is available through the publication by Armstrong et al., 2013). vLFP data were analyzed off-line using a combination of blinded manual seizure identification using custom software (a previous version of which is also available through the publication by Armstrong et al., 2013) and newly generated software using automated algorithms for seizure quantification and flagging of likely behavioral seizures (RRID:SCR_016344; see Code accessibility and Extended data sections).

10.1523/ENEURO.0158-18.2018.ed1 Extended Data 1 Automated ictal spike counter. We have provided the code (created in Matlab 2014b on a Windows 7 computer) to run our custom automated spike counter, used in this article to quantify electrographic seizures. The code runs as a Matlab script, which launches a customizable user interface. A detailed user guide accompanies the code. A copy of the code and user guide can also be found at the URL listed in the *Methods – Code Accessibility* section. Download Extended Data 1, ZIP file.

To ensure robust quantification of electrographic seizures, only a subset of animals with high signal-to-noise recordings underwent analysis (12 dKA subjects; 11 vKA subjects; 10 saline subjects). Electrographic seizures were quantified using an automated algorithm set to customized parameters for each subject. These parameters were based on the mean amplitude of the hippocampal recordings, ictal spike width, and time between spikes. For all animals, a seizure was defined as at least four spikes of greater than twice the baseline amplitude within each 2 s bin. Interictal periods were set to a minimum of 1 s. The minimum seizure duration was originally set at 3 s. In separate analyses, to specifically examine “hippocampal paroxysmal discharges,” event criteria were set to a minimum frequency of 5 Hz for a minimum duration of 5 s (based on the criteria in a study by Twele et al., 2016b). Following automated analysis, 1 h of data were randomly selected for each animal and were manually scored to verify automated detection. Additionally, data from saline controls were analyzed; the results from saline controls indicate a false-positive rate of approximately one electrographic seizure-like event per 20 h of data. Visual inspection confirmed these to be false positives, which is in agreement with a recent report (Twele et al., 2017). All seizure analysis was performed blinded to experimental group.

Large behavioral seizures (LBSs) were defined as >3 on the extended Racine scale of Pinel and Rovner (1978), as further modified by Krook-Magnuson et al. (2015). A scoring of stage 1–3 involved a sudden change in behavioral state, head nodding, or forelimb clonus, respectively; stage 4 involved rearing, bucking, or clonus while on the stomach; stage 5 involved falling or clonus while on a side; stage 6 involved repeated sequences of rearing and falling, otherwise brief jumps; stage 7 involved violent jumping; and stage 8 involved violent jumping followed by a period of tonus lasting >5 s.

### Code accessibility

The automated electrographic seizure analysis software described in the article and accompanying user guide are freely available on-line at https://github.com/KM-Lab/Electrographic-Seizure-Analyzer. These items are also available as Extended Data.

To flag events that were likely to include a behavioral manifestation (i.e., LBSs), a semiautomated approach was used. Raw LFP data were processed using a fast Fourier transform, bandpass filtered between 20 and 200 Hz, reverse fast Fourier transformed, passed through a Butterworth filter, and then smoothed; if the resulting output was greater than a threshold value customized for each subject, that section of data was manually reviewed for the verification of LBS occurrence and scoring of the LBS. This approach correctly flagged all LBSs in a testing dataset that had been previously manually identified, and on at least one occasion, flagged an LBS that escaped manual detection. The data were processed using the following Matlab (RRID:SCR_001622) transformations (with “data” being an array of data and “fs” being the sampling frequency):

fdata = fft(data);

fdata(1:floor(20/fs))=0;

fdata(floor(200/fs):end)=0;

envelope_data = abs(ifft(fdata));

[c,d]butter(2,.1/500,'low');

smooth_envelope_Data = filtfilt(c,d,envelope_data);

### Hyperactivity and anxiety

Several days after the end of vLFP monitoring, animals were subjected to behavioral testing. Animals first performed an open field (OF) and elevated plus maze (EPM) test (mean ± SD: OF: dKA = 54 ± 29 d postinjection; vKA = 57 ± 35 d postinjection; saline = 40 ± 12 d postinjection; EPM: dKA = 59 ± 31 d postinjection; vKA = 63 ± 38 d postinjection; saline = 44 ± 13 d postinjection). The OF apparatus was a 50 × 50 × 40 cm (length × width × height) plastic box. Animals were allowed to roam freely for 15 min. ANY-maze software (Stoelting) was used to record and quantify movement. A 10 cm border around the edge of the apparatus delineated the center of the open field and was used to calculate thigmotaxis. The total distance traveled was also analyzed. In the open field, one subject remained largely immobile throughout testing and was excluded from analysis.

The elevated plus maze (Med Associates) arm dimensions were 34 × 7 cm, at a height of 90 cm. Two of the four arms of the maze were enclosed with tall walls, while the other two arms were open. Animals were placed in the center of the maze and allowed to explore for 5 min. Movement was tracked and analyzed using Ethovision software (Noldus). The time in closed arms and open arms was calculated, in addition to the total distance traveled. Three subjects that fell from the open arms of the elevated plus maze were excluded from analysis. Additionally, one subject was excluded due to improper apparatus setup and another for remaining largely immobile throughout testing.

For both open field and elevated plus maze, testing was performed in dim conditions, ∼50 lux at the center of the maze. On both tests, the arena was cleaned with 70% ethanol between subjects. Subjects were tested by experimenters blinded to experimental condition.

### Cognitive and affective characterization

Object location memory (OLM) and object recognition memory (ORM) testing occurred following OF and EPM testing (mean ± SD: OLM: dKA = 71 ± 27 d postinjection; vKA = 81 ± 27 d postinjection; saline = 66 ± 17 d postinjection; ORM: dKA = 74 ± 16 d postinjection; vKA = 77 ± 29 d postinjection; saline = 63 ± 14 d postinjection). The order of the two tests was alternated between subjects, and there was 1 week between the end of one testing session and the beginning of habituation for the next memory test. The design of the tests was based on previously published protocols (Leger et al., 2013; Vogel Ciernia and Wood, 2014). Mice were handled for 5 d before beginning habituation, once a day for 90 s. After handling, mice were habituated to the OLM or ORM arena twice a day for 5 min at a time, for 3 consecutive days. The following day, two identical objects were introduced to the arena. Subjects were placed into the arena and allowed to explore for 10 min. The following day, two objects were again put into the arenas: for the OLM, object identity remained the same from the previous day, but the location of one object was moved. For the ORM, both objects were put in the same location as the previous day, while one object was changed to a novel object. Subjects were allowed to explore for 5 min. The OLM arena was a rectangular [43 × 22 × 20 cm (length × width × height)] arena painted black, with a white stripe 4 cm in diameter at the center of a long side. The ORM arena was a circular (diameter, 30 cm; height, 26 cm) white arena with a 4 cm black stripe. A thin layer of bedding material covered the floor of the ORM arena. Training and testing sessions were recorded and manually analyzed off-line for bouts of object exploration. A discrimination index (DI) was calculated as follows: difference in time spent investigating novel and familiar objects divided by the sum of all object exploration time. Investigation was defined as the orientation of the subject’s nose directly toward the object and within 1 cm of the edge of the object, exclusive of active engagement in a different activity (e.g., digging). To ensure the consistency of scoring, analysis was performed by two experimenters blinded to the experimental condition. A pre-established exclusion criteria was set at <3 s of total object exploration during the testing session or biased performance during the training session, defined as the absolute value of the DI (using an arbitrary novel object designation) >20. In total, 5 ORM subjects (1 dKA, 1 vKA, 2 saline) and 10 OLM subjects (4 dKA, 4 vKA, 2 saline) were excluded. After finishing the first memory test, subjects were housed in a custom cage for the sucrose consumption task (SCT; described below) before being returned to standard housing for the second memory test.

SCT testing occurred on average 69 d postinjection (mean ± SD: dKA = 74 ± 23 d postinjection; vKA = 82 ± 26 d postinjection; saline = 63 ± 13 d postinjection). Following a published protocol (Katz, 1982; Klein et al., 2015), animals had access to two identical water bottles in a custom cage. After habituating the animal to the cage and water bottles for 3 d, both water bottles were refilled: one with drinking water and the other with a 4% sucrose water solution. Approximately 24 h later, the bottles were both replaced with drinking water. This pattern was repeated for a total of 2 d with access only to drinking water, and 2 d with access to a sucrose solution. The side of the sucrose-containing bottle was counterbalanced across days. Before refilling, bottles were weighed to analyze consumption from the previous day. After refilling, bottles were weighed to set a baseline for the next day. Collection and analysis of SCT data were performed blinded to experimental condition.

### Tissue processing

To assess histopathology, hippocampi ipsilateral to kainate or saline injection were analyzed in coronal sections, comparing MFS and GCD near the dorsal site (∼1.8 mm posterior to bregma) and near the ventral site (∼3.4 mm posterior to bregma). For examination of MFS, Timm staining was performed using modifications of a published protocol (Sunnen et al., 2011). A subset of animals was killed by cervical dislocation (average ± SD age postinjection: dKA, 235 ± 136 d; vKA, 212 ± 150 d; saline, 63 ± 2 d) and transcardially perfused with ∼30 ml of 0.9% saline, 50 ml of 0.37% sodium sulfide, 20 ml of 0.9% saline, and 50 ml of 4% PFA. Brains were then postfixed for 48 h in 4% PFA. Brains were sectioned at 50 μm and were processed for Timm staining. Timm stain consisted of an ∼60% arabic gum solution; a 10% 2 m, pH 4, citrate buffer; 30% 0.5 m hydroquinone; and <1% of a 19% silver nitrate solution. Slices were mounted on a slide and dried for 3 h at room temperature, then submerged in the Timm stain for 45 min at room temperature. Slides were then rinsed with water and submerged in 5% sodium thiosulfate for 5 min. Slides were then dehydrated in a series of ethanol baths and mounted with DPX Mountant (Sigma-Aldrich). Light microscopy was used to capture images of the dentate gyrus. A region of interest was drawn around the granule and molecular layers, which were thresholded to capture blackened (Timm-stained) tissue and were expressed as a fraction of the total area of the molecular and granule cell layers. Observations were taken from two hippocampal sections per animal (one anterior, one posterior).

Another series of brain slices was collected from a subset of animals and mounted on slides with DAPI (4′,6′-diamidino-2-phenylindole dihydrochloride) mounting media (Vectashield; average age postinjection, 142 d; mean ± SD: dKA = 186 ± 134 d postinjection; vKA = 148 ± 136 d postinjection; saline = 100 ± 55 d postinjection). As with MFS, observations for GCD were taken from two hippocampal sections per animal (one anterior, one posterior). The width of the widest point of the central two-thirds of the enclosed blade of the dentate gyrus ipsilateral to injection was measured to assess GCD. The boundaries of the granule cell layer were defined to end when there was a >20 µm gap between nuclei. Image quantifications were performed using ImageJ (RRID:SCR_002285). All tissue processing and analysis was performed blinded to experimental condition.

### Statistical analysis

Statistical analysis was conducted using OriginPro 2016. Where indicated, power analyses were performed using G*Power 3.1 with observed means and SDs. Unless stated otherwise, values are reported as the mean ± SEM. A *p* value of <0.05 was considered significant. Though the experiments were not designed to test for sex differences, no significant differences were observed between male and female subjects.

## Results

### Examination of anatomic pathologies

**Figure 1.**
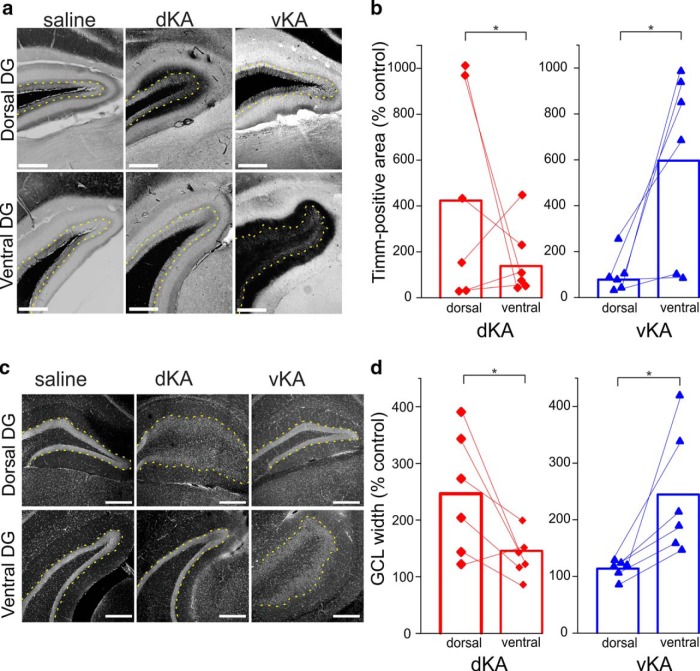
Similar anatomic pathologies maximally present near site of KA injection. ***a***, Timm-stained images of the dentate gyrus (DG) of saline, dKA, and vKA animals near the area of dorsal and ventral targeting. Yellow dotted lines indicate the boundary of the granule cell layer and inner molecular layer. Scale bar, 300 µm. ***b***, Comparison of Timm-positive granule cell and molecular layer area, normalized to saline controls, between dorsal and ventral hippocampal locations for dKA and vKA groups (dKA, *n* = 6 animals; vKA, *n* = 6 animals). ***c***, DAPI-stained images of dentate gyri from saline, dKA, and vKA animals. Yellow dotted lines indicate the boundary of the granule cell layer and inner molecular layer. Scale bar, 300 µm. ***d***, Comparison of granule cell layer width, normalized to saline controls, at dorsal and ventral locations for dKA and vKA animals (dKA, *n* = 6 animals; vKA, *n* = 6 animals). *p < 0.05. Dorsal location, ∼1.8 mm past bregma; ventral location, ∼3.4 mm posterior to bregma. Red bar and data points reflect dKA data; blue bar and data points reflect vKA data.

Epilepsy can present with histologic, electrophysiological, cognitive, and affective pathologies. We therefore sought to characterize the vKA model, in which kainic acid is injected into the ventral hippocampus, in addition to the standard dKA model, in which kainic acid is injected into the dorsal hippocampus, along these various phenotypes. In both human patients and animal models of temporal lobe epilepsy, including the dKA model, anatomic signs of epileptic pathology in the tissue of the temporal lobe are often present. In addition to hippocampal sclerosis (Bouchet and Cazauvieilh, 1825; Andersson et al., 1991; Suzuki et al., 1995; Bouilleret et al., 1999; Arabadzisz et al., 2005), aberrant sprouting of mossy fibers (i.e., MFS; Fig. 1*a*) as well as dispersal of the dentate gyrus granule cell layer (i.e., GCD; Fig. 1*c*) are classical histopathologies (Scheibel et al., 1974; Houser,1990). To determine whether and where MFS and GCD manifest in the vKA model, we analyzed postmortem tissue. For GCD, we analyzed the width of the granule cell layer. For MFS, we analyzed the amount of Timm-stained tissue in the granule and molecular layer of the dentate gyrus. Both groups exhibited differences in MFS between the respective site of kainate injection compared with the corresponding uninjected site, supporting the distinction between the two sites of KA injection. For the dKA group, MFS was greater in the dorsal compared with the ventral hippocampus (Wilcoxon signed rank test: *n* = 6 animals, *p* = 0.036; mean at dorsal site saline control, 422 ± 172%; mean at ventral site saline control, 137 ± 60%). For the vKA group, the reverse was true: MFS was greater at the ventral site compared with the dorsal site (Fig. 1*b*; Wilcoxon signed rank test: *n* = 6 animals, *p* = 0.036; mean at dorsal site saline control, 77 ± 31%; mean at ventral site saline control, 596 ± 156%). Results from examining GCD followed a similar pattern (Fig. 1*d*). In the dKA group, GCD was greater in the dorsal aspect of the hippocampus compared with the ventral aspect (Wilcoxon signed rank test: *n* = 6 animals, *p* = 0.036; dorsal site mean saline control, 246 ± 41%; ventral site mean saline control, 144 ± 14%). Likewise, for the vKA group, the GCD was greater in the ventral compared with dorsal hippocampus (Wilcoxon signed rank: *n* = 6 animals, *p* = 0.036; mean at dorsal site saline control, 113 ± 5%; mean at ventral site saline control, 244 ± 41%). These data indicate that vKA and dKA models both produce anatomic pathologies but that the models exhibit different pathologic focal points corresponding to their respective site of kainate injection. These histopathological results support the distinction of the two sites of KA administration.

### Electrographic seizures

Given the differing sites of maximal anatomic pathology, we investigated whether the dKA and vKA insults generate different seizure phenotypes. At least 2 weeks after KA or saline injection, animals were placed under chronic video-synchronized LFP monitoring. Hippocampal activity was recorded via a twisted wire electrode placed approximately equidistant between the two sites of KA injection. This setup continuously recorded video and hippocampal LFP data for 2 weeks. The data were analyzed off-line by a combination of blinded manual and semiautomated analyses. In total, 7943 h of data were analyzed for electrographic seizures.

**Figure 2.**
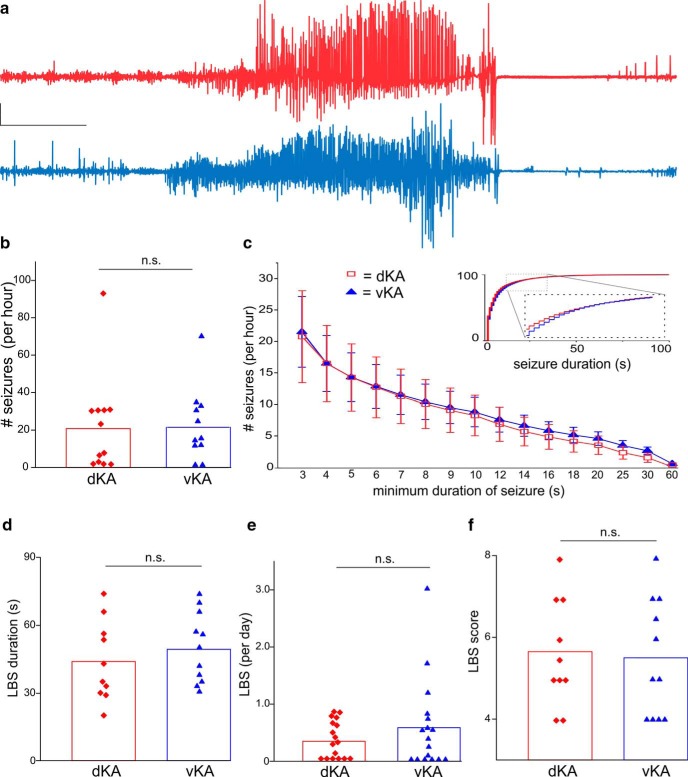
dKA and vKA groups exhibit similar electrographic and LBS burdens. ***a***, Recordings from dKA (top) and vKA (bottom) animals during an LBS. Calibration: 200 µV, 10 s. Large-amplitude signals are truncated. ***b***, Quantification of electrographic seizure frequency for seizure events with a minimum duration of 3 s. ***c***, Distribution of the frequency of electrographic seizure by duration. Inset, Cumulative distribution of electrographic seizures rerecorded from a subset of mice at a later time point. Note the similar distributions of seizure durations between dKA and vKA also at this later time point. ***d***, Comparison of the average duration of LBS events. ***e***, Frequency of LBSs. ***f***, Comparison of median LBS scores. Red quadrilaterals reflect individual dKA data points; blue triangles reflect vKA individual data points. Error bars in ***c*** indicate SEM. n.s., Nonsignificant.

Examination of electrographic seizures revealed similar frequencies in both the dKA and vKA groups when a seizure was defined to have a minimum 3 s duration (Fig. 2*b*; Mann–Whitney test, *p* = 0.78; dKA: *n* = 12 animals; mean, 20 ± 7 events/h; vKA: *n* = 11 animals; mean, 21 ± 5 events/h). To ensure that the lack of difference was not due to insufficient power, a power analysis was performed, which indicated that >300 subjects (150 dKA and 150 vKA) would be needed to demonstrate a statistically significant result.

Comparing the rates of seizures with varying minimum durations between dKA and vKA groups further failed to reveal any differences (uncorrected Mann–Whitney *p* values > 0.17 for all examined minimum seizure duration definitions; Fig. 2*c*). This finding indicates a similar seizure burden between the two groups, regardless of the minimum duration chosen.

Further analysis of the data to specifically examine the occurrence of hippocampal paroxysmal discharge-like events (requiring a minimum spike frequency of 5 Hz and a minimum duration of 5 s; based on the study by Twele et al., 2016b) again failed to reveal significant differences between dorsal and ventral groups (Mann–Whitney ANOVA, *p* = 0.35; dKA: *n* = 12 animals; mean, 4.4 ± 2 events/h; vKA: *n* = 11 animals; mean, 7.3 ± 2 events/h).

An additional analysis was performed examining seizures not at the subject level but at the seizure level. Examination of electrographic seizure durations described only a small (0.6 s) difference between dKA and vKA seizures (Kolmogorov–Smirnov test, *p* < 0.001; dKA: *n* = 37,320 seizures; median duration, 7.9 s; vKA: *n* = 49,272 seizures; median duration, 7.3 s).

To test whether a more substantial difference between groups would be apparent at a later time point, a subset of mice were rerecorded months later. Not all animals survived to this later time point, but mortality levels were similar between dKA and vKA groups (Fisher’s exact test, *p* > 0.9; dKA: mortality, 4 of 17 animals; vKA mortality, 5 of 17 animals). Examination of electrographic seizure durations at this later time point again suggested only a small (0.6 s) difference between dKA and vKA seizures (Kolmogorov–Smirnov test, *p* < 0.001; dKA: *n* = 16,875 seizures; median duration, 4.9 s; vKA: *n* = 15,601 seizures; median duration, 5.5 s; Fig. 2*c*, inset), indicating that a lack of substantial difference was not due to the time point examined.

These data indicate that overall seizure burden is remarkably similar between dKA and vKA models at both an earlier and a later time point, suggesting that targeting the dorsal and ventral hippocampus for initial epileptogenic insults results in approximately equal ictogenicity in chronic epilepsy.

### Large behavioral seizures

In contrast to the high frequency of electrographic seizures, ictal events with a large behavioral component (i.e., LBS) occurred much less frequently. Analysis indicated similar LBS burdens between dKA and vKA animals, with no significant differences in frequency (Mann–Whitney test, *p* = 0.78; dKA: *n* = 17 animals; mean LBSs/d, 0.35 ± 0.08; vKA: *n* = 17 animals; mean LBSs/d, 0.59 ± 0.2), duration (*p* = 0.36; mean ± SD: dKA, 44 ± 5 s; vKA, 49 ± 4 s), or median seizure severity (*p* = 0.75; dKA, 5.25; vKA, 5; Fig. 2*d–f*). To again ensure that the lack of difference was not due to insufficient power, an additional power analysis was performed, which suggested that almost 400 subjects (200 dKA and 200 vKA) would be needed to demonstrate a statistically significant result.

An additional analysis was performed examining seizure duration and severity not at the subject level but at the seizure level. While there was a small (0.2 s) difference in LBS duration (Mann–Whitney test, *p* = 0.0252; dKA: *n* = 80 LBSs; mean, 49.4 ± 3.3 s; vKA: *n* = 126 LBSs; mean, 49.2 ± 1.4 s), seizure severity remained similar (Mann–Whitney test, *p* = 0.13; dKA: *n* = 80 LBSs; median, 5; vKA: *n* = 126 LBSs; median, 5). Therefore, for both electrographic and large behavioral seizures, no substantial differences in seizure burden were found between dKA and vKA models.

### Cognitive and affective characterization

Despite having similar seizure rates, we wondered whether the dKA and vKA models would present distinct cognitive or affective phenotypes. Given that seizure burdens were similar between the dKA and vKA groups, any differences in behavior or affect would be more directly related to the site of KA injection, instead of secondary to effects of differences in seizure burden. We challenged the mice with tests of spatial and nonspatial memory (OLM and ORM, respectively), as well as tests of hyperactivity, anxiety (OF, EPM), and anhedonia (SCT).

**Figure 3.**
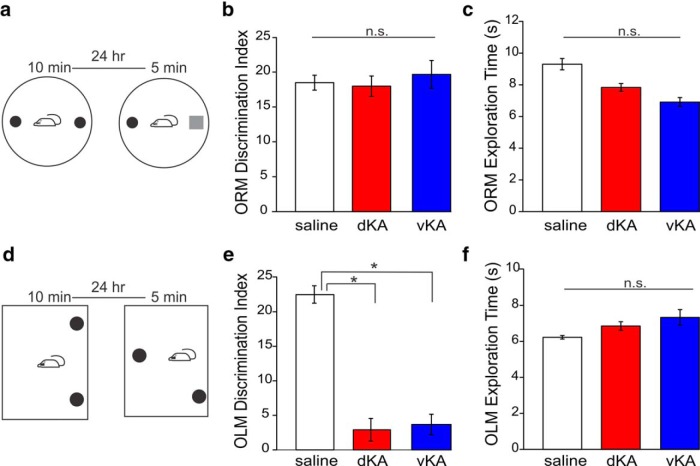
Spatial but not object identity memory deficits are present in both dKA and vKA models. ***a***, Schematic of the training and testing portion of the ORM test. ***b***, Comparison of novel object discrimination ability between saline, dKA, and vKA groups (saline, *n* = 13 animals; dKA, *n* = 12 animals; vKA, *n* = 11 animals). ***c***, Combined object exploration time on the ORM test. ***d***, Schematic of the training and testing portion of the OLM test. ***e***, Comparison of novel location discrimination ability among the saline, dKA, and vKA groups (saline, *n* = 13 animals; dKA, *n* = 8 animals; vKA, *n* = 10 animals). ***f***, Combined object exploration time on the OLM test. *p < 0.05. n.s., Nonsignificant difference. Open bars indicates saline data; red bars reflect dKA data; blue bars reflect vKA data. Error bars indicate SEM.

The OLM test is known to be sensitive to hippocampal dysfunction, while the ORM test shows less sensitivity to hippocampal damage (Mumby et al., 2002; Winters et al., 2004; Winters and Bussey, 2005; Balderas et al., 2008; Haettig et al., 2011). On the ORM, a nonspatial memory test, all groups exhibited similar abilities to discriminate a novel object (Kruskal–Wallis ANOVA, *p* = 0.74; saline: *n* = 13 animals; mean DI, 19.3 ± 1.2; dKA: *n* = 12 animals; mean DI, 18 ± 1.5; vKA: *n* = 11 animals; mean DI, 19.7 ± 2; Fig. 3*b*). These results indicate that object–identity memory is uncompromised in our dKA and vKA groups.

Previous reports have observed deficits for dKA animals on the OLM test (Bui et al., 2018). We investigated whether the vKA group exhibits similar spatial memory deficits on the OLM task. Both the dKA and vKA groups demonstrated poor spatial novelty discrimination relative to saline-treated controls (Kruskal–Wallis ANOVA, *p* = 0.011; saline: *n* = 13 animals; mean DI, 22.5 ± 1.2; dKA: *n* = 8 animals; mean DI, 2.9 ± 1.6; vKA: *n* = 10 animals; mean DI, 3.6 ± 1.5; Mann–Whitney test: dKA vs saline, *p* = 0.012; vKA vs saline, *p* = 0.014; dKA vs vKA, *p* = 0.96; Fig. 3*e*). These effects cannot be explained by differences in motor or motivational domains for several reasons. First, subjects were capable of discrimination on the ORM, revealing that they were competent to investigate novelty. Furthermore, dKA, vKA, and saline groups spent similar amounts of combined time investigating novel and familiar objects, indicating similar object engagement (Fig. 3*c,f*; OLM: Kruskal–Wallis ANOVA, *p* = 0.79; ORM: Kruskal–Wallis ANOVA, *p* = 0.48). These data therefore reveal that dKA and vKA mice possess similar memory deficits that are sensitive to spatial memory function without impairments of more generalized, object identity memory.

**Figure 4.**
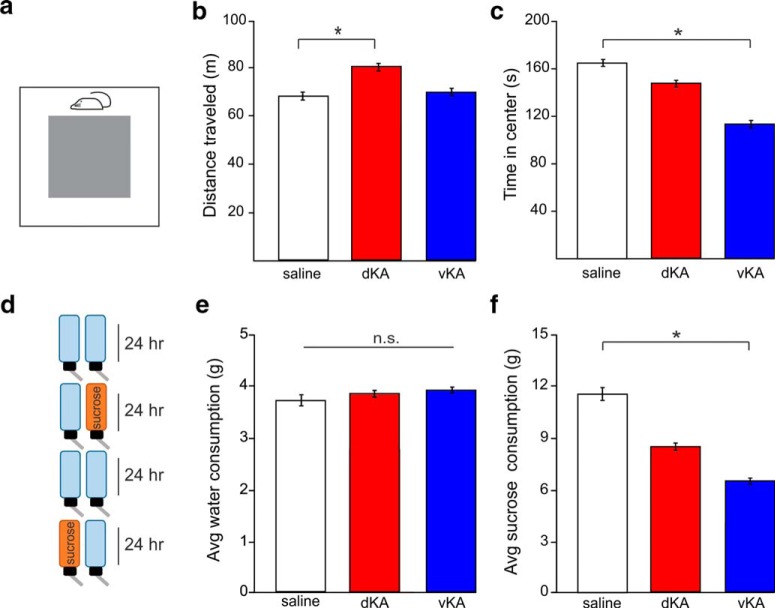
Hyperactive, anxiety, and anhedonic phenotypes are differentially present in dKA and vKA models. ***a***, Schematic of the open field test. ***b***, Comparison of distance traveled during the open field test, a measure of hyperactivity (saline, *n* = 15 animals; dKA, *n* = 13 animals; vKA, *n* = 14 animals). ***c***, Time spent in the center of the open field arena, a negative indicator of anxiety. ***d***, Schematic of SCT. ***e***, Comparison of average amount of water consumed on days with exposure to only water (saline, *n* = 16 animals; dKA, *n* = 12 animals; vKA, *n* = 13 animals). ***f***, Comparison of average amount of sucrose water consumed when sucrose water was available, a test of anhedonia. *p < 0.05. n.s., Nonsignificant difference. Open bars indicates saline data; red bars reflect dKA data; blue bars reflect vKA data. Error bars indicate SEM.

In addition to spatial memory deficits, we asked whether either the dKA or vKA groups exhibit affective phenotypes. Two tests were used to examine hyperactivity and anxiety: the OF and EPM. In the open field, the dKA group traveled a greater distance than the saline group. In contrast, however, the vKA group did not exhibit this phenotype (Kruskal–Wallis ANOVA, *p* = 0.041; saline: *n* = 15 animals; mean distance, 66.4 ± 0.7 m; dKA: *n* = 13 animals; mean distance, 80.8 ± 1.2 m; vKA: *n* = 14 animals; mean distance, 69.7 ± 0.9 m; Mann–Whitney ANOVA: dKA vs saline, *p* = 0.017; Mann–Whitney test: vKA vs saline, *p* = 0.42; Fig. 4*a* and 4*b*).

When examining the time in the center of the open field (as a measure of anxiety), there was a strong trend in overall difference between groups (Kruskal–Wallis ANOVA: *p* = 0.062; saline: mean duration, 164.3 ± 2.8 s; dKA: mean duration, 151.9 ± 2.9 s; vKA: mean duration, 114 ± 4.4 s) and individual comparisons exposed a significant difference between the vKA and saline groups (Mann–Whitney test, *p* = 0.024) but not between the dKA and saline groups (Mann–Whitney test, *p* = 0.58; Fig. 4*c*).

Repeated testing of these phenotypes in a different experimental paradigm, the elevated plus maze, suggested a similar increase in dKA distance traveled (supporting a hyperactive phenotype in the dKA model), while vKA animals trended toward this (Kruskal–Wallis ANOVA, *p* = 0.043; saline: *n* = 14 animals; mean distance, 21.6 ± 0.3 m; dKA: *n* = 11 animals; mean distance, 25.5 ± 0.5 m; vKA: *n* = 10 animals; mean distance, 26.2 ± 0.8 m; Mann–Whitney test: dKA vs saline, *p* = 0.018; Mann–Whitney text: vKA vs saline, *p* = 0.1). However, data from the elevated plus maze did not show any between-group difference in time spent in open arms of the maze—another measure of anxiety (Kruskal–Wallis ANOVA: *p* = 0.84; saline: mean duration, 107 ± 14 s; dKA: mean duration, 115 ± 18 s; vKA: mean duration, 103 ± 23 s). Together, these data from the open field and elevated plus maze experiments indicate a hyperactive phenotype present in the dKA animals while suggesting a possible anxiety phenotype in the vKA animals.

In another series of experiments, a different affective pathology was assessed: anhedonia. Anhedonia is a key symptom of clinical depression, which is highly comorbid in individuals with TLE (Scott et al., 2017) and impairs quality of life (Johnson et al., 2004). We used the SCT to determine whether either the dKA or vKA group freely participated in a pleasurable activity (consuming sucrose water) at comparable levels to those of saline controls. When only standard water was available, there was no difference in water consumption among groups, indicating a similar propensity and ability to engage in drinking behavior (Kruskal–Wallis ANOVA, *p* = 0.99; saline: *n* = 16 animals; mean, 3.8 ± 0.1 × *g*; dKA: *n* = 12 animals; mean, 3.9 ± 0.1 × *g*; vKA: *n* = 13 animals; mean, 3.9 ± 0.1 × *g*; Fig. 4*e*). Examination of sucrose water consumption revealed a group difference (Kruskal–Wallis ANOVA, *p* = 0.022; saline: mean, 11.5 ± 0.7 × *g*; dKA: mean, 8.7 ± 0.4 × *g*; vKA: mean, 6.6 ± 0.3 × *g*) in which vKA animals drank less sucrose water than saline animals (Mann–Whitney, *p* = 0.003). The dKA group appeared to show a more intermediate phenotype; analysis of consumed sucrose water trended toward reduced consumption (Mann–Whitney test: dKA vs saline, *p* = 0.086; Fig. 4*f*). These data highlight the potential for the vKA model of TLE to emphasize specific affective comorbidities of TLE, as the difference in sucrose water consumption suggests a pronounced anhedonic phenotype in the vKA group.

## Discussion

The vKA model successfully captures key elements of TLE pathology that make the standard dKA model successful. Specifically, our data demonstrate that the vKA model exhibits classical anatomic pathologies, generates spontaneous electrographic and behavioral seizure burdens similar to those of the dKA model, and presents hippocampal-specific cognitive deficits. Importantly, the vKA model additionally displays a pronounced affective phenotype that includes anhedonia. These results indicate that the vKA model of TLE may be better suited for studying affective comorbidities of human TLE than the standard dKA model.

Where the dKA and vKA groups differ the most is in their affective and hyperactivity phenotypes. Specifically, the vKA group exhibited a strong anhedonic phenotype in addition to a potential anxiety phenotype, whereas the dKA group only exhibited significant hyperactivity. A recent meta-analysis estimated the prevalence of depressive disorders in people with epilepsy to be 23% (Scott et al., 2017), making it the most frequently comorbid psychiatric disorder in epilepsy (Kanner, 2003). Depression is known to have a major negative effect on quality-of-life measures in people with TLE (Johnson et al., 2004) and is repeatedly reported to be a more powerful predictor of quality-of-life scores than seizure frequency (Perrine et al., 1995; Gilliam et al., 1997; Boylan et al., 2004). The prevalence and impact of this comorbidity highlights its importance in patients with TLE and underscores the necessity to recreate depressive phenotypes in animal models. Our data indicate that the vKA model is better suited for the study of anhedonia than the standard dKA model, which only trended toward reduced sucrose consumption. It is worth noting that a previous report found significant anhedonia in the dKA model (Klein et al., 2015), suggesting that the dorsal model can show some degree of anhedonia. However, our data show that ventral hippocampal KA administration strongly elicits anhedonia. While depressive-like behavior has been reported in various TLE models (Mazarati et al., 2008, 2009, 2010; Tchekalarova et al., 2011; Vrinda et al., 2017), it has not been universally observed. In fact, multiple reports indicate a decrease in measures of depressive-like behavior in various TLE models (Wintink et al., 2003; Adamec et al., 2004; Dos Santos et al., 2005; Inostroza et al., 2012; Lopes et al., 2016). The mechanisms underlying these disparate findings require further investigation to be fully understood, but suggest that lesion location, rather than the presence of seizures per se, may play a role in affective comorbidities. Our findings highlight the potential usefulness of the vKA model to study comorbid depression, which remains a substantial burden to the human TLE population.

Similar to depression, anxiety is also highly comorbid and detrimental to people with TLE, with an estimated prevalence of 20% (Scott et al., 2017) and a significant negative impact on quality-of-life scores (Johnson et al., 2004). The relationship between the ventral hippocampus and depression as well as anxiety is well documented outside the epilepsy field. Ventral hippocampal afferents to the nucleus accumbens have been shown to regulate susceptibility to depression using a chronic social defeat paradigm (Bagot et al., 2015). Furthermore, ventral hippocampal efferents were found to be critical for the antidepressant effect of ketamine (Carreno et al., 2016). Additionally, lesion studies have demonstrated that the ventral, but not dorsal, hippocampus is required for normal anxiety behaviors (Bannerman et al., 2004), and *in vivo* electrophysiological studies have demonstrated that coordination between the prefrontal cortex and ventral, but not dorsal, hippocampus is synchronized during anxiety (Adhikari et al., 2010). The disruption of normal ventral hippocampal networks following KA administration could impact these networks, mediating affective behavior, thereby generating anhedonic and possible anxiety phenotypes.

Our data present some evidence for a potential anxiety phenotype in the vKA model, as there was a significant reduction in the time spent in the center of an open field test. However, no difference was seen on the open arms of the elevated plus maze, another classic test for anxiety. This suggests either a task-specific anxiety or only a mild anxiety phenotype in the vKA animals. Similarly, an effect in the open field but a lack of an effect in the elevated plus maze has been previously observed in the pilocarpine model of TLE (Gröticke et al., 2007). Notably, no strong evidence for an anxiety phenotype was seen for dKA animals in the present study.

Similar to reports on depressive-like behavior, the literature on anxiety in animal models of TLE is highly mixed. Across different rodent species, different timepoints, and different TLE models (e.g., dKA, intraperitoneal pilocarpine, intraperitoneal KA, amygdalar kindling, amygdalar KA), some studies report increased anxiety-like behavior (Wintink et al., 2003; Mohajeri et al., 2004; Müller et al., 2009; Liu et al., 2013; Lopes et al., 2016; Otsuka et al., 2016), while others report decreased anxiety-like behavior (Adamec et al., 2004; Detour et al., 2005; Dos Santos et al., 2005; Inostroza et al., 2011, 2012; Vrinda et al., 2017). Differences in methods and models, as well as potentially in strains and species, undoubtedly play a role in the controversy (Klee et al., 2017; Löscher et al., 2017). As with depression, the expression of anxiety comorbidities may be strongly influenced by lesion location, especially as it relates to amygdalar circuits (Inostroza et al., 2011, 2012). The amygdala itself is a target for focal induction of TLE, including by administration of KA (Ben-Ari et al., 1979, 1980; Hasegawa et al., 2002; Mouri et al., 2008; Liu et al., 2013). Anxiety-like behavior in intra-amygdala KA-injected mice has been observed and was ameliorated by post-status epilepticus intervention (Liu et al., 2013), suggesting the observed anxiety phenotype was sensitive to ongoing epileptic processes. However, other reports using systemic chemovulsants in rats noted anxiolytic—rather than anxiogenic—effects occurring alongside amygdalar damage (Inostroza et al., 2012). A comprehensive explanation of the differences in anxiety phenotypes currently evades the field, and additional research is needed to fully understand the underlying mechanisms of anxiety in models of epilepsy.

Tissue from human patients with TLE often displays both GCD and MFS (Scheibel et al., 1974; Houser, 1990). In the dKA and vKA models, we observe the presence of these pathologies and additionally identify the maximal site of pathology to be near the site of KA injection in each model. These data support the distinction of the dKA and vKA models and suggest that the functional effects of these pathologies would be mostly localized to either the dorsal or ventral hippocampus, respectively. This would then have differential effects on hippocampal function and behavior. However, the overall network contribution of MFS and GCD is not fully understood. Some evidence suggests that MFS may be excitatory and facilitate seizures via recurrent granule cell excitation (Wuarin and Dudek, 1996; Lynch and Sutula, 2000; Feng et al., 2003; Scharfman et al., 2003). Other data suggest that MFS may provide inhibitory effects via synapses on inhibitory neurons or the release of inhibitory transmitters (Sloviter, 1992; Schwarzer and Sperk, 1995; Tu et al., 2005; Sloviter et al., 2006). To further complicate the issue, MFS has been reported both to be correlated with seizure frequency (Wenzel et al., 2000; Kharatishvili et al., 2006; Hester and Danzer, 2014) and to be dissociated from seizure frequency (Zhang et al., 2002; Williams et al., 2004; Buckmaster and Lew, 2011). In a similarly unresolved fashion, the role of GCD in the altered hippocampal network remains uncertain. Some evidence suggests decreased excitability of dispersed granule cells (Young et al., 2009), but the contribution of this effect to epileptic pathology and aberrant network processing requires additional investigation. Regardless of the precise effects that MFS and GCD have on the hippocampal network, our data suggest that those effects would be magnified at different parts of the hippocampus in the dKA and vKA models.

Given the differences in anatomic pathology, and the known differences between the dorsal and ventral aspects of healthy and epileptic hippocampi, we were surprised to observe no substantial differences between the dKA and vKA groups in their ictal phenotypes. Seizure frequency and duration, for both electrograph and LBS, were remarkably similar between groups, as was LBS severity. In contrast, several lines of evidence from the epilepsy field describe differences between dorsal and ventral portions of the hippocampus in terms of their ictogenicity. First, in an observational study of patients with TLE, the human anterior hippocampus (corresponding to the rodent ventral hippocampus) was found to be more ictogenic than the posterior hippocampus (corresponding to the rodent dorsal hippocampus; Engel et al., 1990). Second, *in vitro* work observed that the rodent ventral hippocampus generates more frequent spontaneous seizure-like bursting than the dorsal hippocampus (Bragdon et al., 1986). Furthermore, data from animal models suggest that a population of ventral, but not dorsal, hippocampal granule cells is necessary for wet-dog shakes following acute seizure induction (Grimes et al., 1990). In a similar vein, an early kindling study demonstrated that kindling of the ventral hippocampus more quickly produced generalized seizures than the dorsal hippocampus (though the ventral hippocampus also had a greater afterdischarge threshold; Racine et al., 1977). More recently, a study using optogenetics to induce seizures found that seizures generated in the dorsal hippocampus were less likely to generalize than seizures initiated in a more ventral (intermediate) region (Weitz et al., 2015). Together, these studies support an important distinction between dorsal and ventral hippocampal areas in both healthy function and ictal pathology.

The finding that the dKA and vKA models exhibit similar rates of both electrographic and large behavioral seizures in chronic epilepsy raises several interesting prospects. For one, differences in ictal frequency in acute studies may not generalize to chronic models of spontaneous seizures. Consider, for example, the increased current injections required for afterdischarges in kindling targeting the ventral hippocampus (Racine et al., 1977), which suggests an increased initial ictal threshold in the ventral hippocampus. Additionally, during the epileptogenic process, there is substantial reorganization of the hippocampal network, and this reorganization may disrupt the processes that underlie findings of differences in acute seizures between the dorsal and ventral hippocampus. Our results demonstrate that whatever differential changes enacted by MFS and GCD in their respective hippocampal domains of the dKA and vKA models, they were either insufficient to generate a difference in ictal characteristics or were overwhelmed by other changes that overlapped between the two models.

With both dKA and vKA groups exhibiting similar ictal phenotypes, the potential confound of secondary effects of ictal burdens is removed. This is important when evaluating the dKA and vKA models on multiple pathologic dimensions, as seizure burden can influence cognitive and affective outcomes. For example, seizure frequency and severity are known to correlate with progressive cognitive deterioration in people with TLE (Helmstaedter et al., 2003). Additionally, postictal periods are associated with worsened psychiatric problems in human TLE patients (Kanner et al., 2004). The similarity of the ictal phenotypes of the dKA and vKA models allows us to more confidently compare their cognitive and affective pathologies as results from differences in hippocampal targeting instead of differences in seizure burden.

Both the dKA and vKA groups had a similar impairment on a test of hippocampal-dependent spatial memory, without showing impairments on a test of nonspatial memory. Though the dorsal hippocampus is generally associated with spatial navigation and memory, there are several possibilities that could account for the vKA group also showing spatial impairment. For one, following ventral KA injection, broad network changes may impair the ability of the dorsal hippocampus to mediate spatial memory. Alternatively, the ventral hippocampus is known to be involved with some aspects of spatial memory, and damage to these functions could cause the observed deficit. For example, in addition to the dorsal hippocampus, the ventral hippocampus also has place cells, although they generally govern a much larger place field than their dorsal counterparts (Kjelstrup et al., 2008). Additionally, lesion studies suggest that, although encoding of spatial memory requires only the dorsal hippocampus, retrieval of spatial memory may require some aspects of the ventral hippocampus (Moser and Moser, 1998). However, deficits in spatial memory in the dKA and vKA models may also be viewed less as a result of acute hippocampal KA lesion location and more as a consequence of recurrent seizures. Impairment in spatial memory-related processes, including acquisition, recall, and episodic-like memory, has been reported in chronic models of TLE using different systemic chemoconvulsants across multiple species (Gröticke et al., 2008; Chauvière et al., 2009; Inostroza et al., 2011, 2013; Suárez et al., 2012) as well as from acute seizure models (Lin et al., 2009). As seizures themselves may disrupt hippocampal function, it is important to remember that dKA and vKA groups displayed similar seizure burdens. Seizure burden alone, however, is not the sole predictor of cognitive impairment (Inostroza et al., 2011). Regardless of the role that the dorsal and ventral hippocampus play in spatial memory, the phenotype of impaired spatial memory is shared among both the dKA and vKA models, as well as other nonfocal models. Other pathologies, however, such as anhedonia or hyperactivity, seem more influenced by the exact means of epileptogenic induction.

The dKA model benefits from extensive prior characterization, such as the response to antiepileptic drugs (Klein et al., 2015; Duveau et al., 2016) and optogenetic (Krook-Magnuson et al., 2013, 2014, 2015; Bui et al., 2018) and electrical intervention (Bragin et al., 2002; Huang and van Luijtelaar, 2013), characterization of pathological timecourse (Gruber et al., 1994; Bragin et al., 2005; Heinrich et al., 2006, 2011; Häussler et al., 2012; Li et al., 2018), changes in hippocampal activity and circuitry (Davenport et al., 1990; Mathern et al., 1993; Bouilleret et al., 1999; Bragin et al., 1999; Riban et al., 2002; Lu et al., 2016; Christenson Wick et al., 2017; Sheybani et al., 2018), molecular mechanisms (Straessle et al., 2003; Young et al., 2009; Duveau and Fritschy, 2010; Schidlitzki et al., 2017), interactions with the hypothalamic–pituitary–gonadal axis (Li et al., 2016), and effects of strain and sex (Twele et al., 2016a). Future work can similarly further examine the vKA model in regard to these important issues.

Overall, the vKA model of TLE exhibits similar histopathological and ictal phenotypes compared with the standard dKA model, but displays additional affective phenotypes. This model provides a tool for epilepsy researchers to further examine important comorbidities of TLE, such as depression, while maintaining the benefits of a focal KA model.

## Synthesis

Reviewing Editor: Liset Menendez de la Prida, Instituto Cajal CSIC Decisions are customarily a result of the Reviewing Editor and the peer reviewers coming together and discussing their recommendations until a consensus is reached. When revisions are invited, a fact-based synthesis statement explaining their decision and outlining what is needed to prepare a revision will be listed below. The following reviewer(s) agreed to reveal their identity: Carola Haas, Laura Ewell.

Your ms was reviewed by two independent referees and myself. We agreed there is value in your data, however we had some conflicting views regarding acceptance. One reviewer recommended rejection and had serious methodological concerns. I had some questions regarding statistics, data analysis and presentation, while the other reviewer was overall very positive with only some questions. We discussed and agreed you may be able to address the issues with additional analysis and writting requiring less than two months. We therefore agreed on a major revision.

Here is a list of those concerns and comments. I chose to lump them all in a single report. Please, note the order of comments is not informative. You need to adress all of them properly. Your revised version will go to both reviewers again.

-Number of animals for each experiment should be given in the text, and not only at Fig. captions

-I found interesting how authors use power analysis in the narrative. Please, include specific details in methods

-With almost 20 seizures per day, I wonder how informative is cognitive performance?

-The paper is poorly referenced in general. I would discuss a bit more previous characterization of the intra-hippocampal Kainate model and possibly the i.p kainate model. For intance, our data with the ip model show specific cognitive deficits in the abscence of seizures (see Inostroza et al. PLoS ONE 2011, PLoS ONE 2012 and JN 2013). You don't need to cite our papers, I just refer for comparison with the ip model. Also, consider some of the following citations for the intra-hippocampal model: Suzuki et al., 1995 (1st description of iKA model

Riban et al., 2002 (ephys iKA model)

Heinrich et al. JN 2006, 2011(time course of dispersion)

Häussler et al CerCor 2012 (time course of LFP). We also feel it may be useful to dicuss on the intra-amygdala KA model. Consider some of these: G. Mouri, ... D Henscehll 2008(intra-amygdala KA)

G. Liu, ... J McNamara et al 2013 (used the same model, intra-amygdala KA)

-Fig.1: Some images are poor quality and out of focus. Poor statistical differences suggest large dispersion, please show single data points. Please, clarify how are data analyzed (section/animal).

-Figure 1a - vKA, ventral DG - it is difficult to resolve the GC layer versus the IML in this panel. Could you add some dotted lines around the GCL to help guide the reader?

- Figure 1a: The sections of the “dorsal” DG do not represent dorsal hippocampal sections. There is granule dispersion missing in the dorsal DG/dKA section. Figure 1b. The quality of the DAPI-stained sections is rather poor.

-Line 84 - Change “lead” to “led”

-Throughout consider revising the text to say “LFP monitoring” versus “depth EEG”.

-Fig.2: the n here should be seizures. A nested design (seizure/animal) may affect statistical report

-Line 38: “Following a latent period, this model produces spontaneous, recurring seizures as well as classic histopathological features also observed in human TLE: sprouting of mossy fibers (mossy fiber sprouting, MFS) and dispersion of granule cells (granule cell dispersion, GCD) (Bouilleret et al., 1999)”.

-Human mTLE is accompanied by hippocampal sclerosis which is characterized by selective cell death and not only by mossy fiber sprouting and granule cell dispersion, but this feature is not mentioned at all.

-Methods: Why were both sexes used in this study? Usually only males are used for this mTLE model. Please, clarify

-Kainate (KA) injection: here 100nL of 20 mM kainate were injected. This is twice the volume and amount which is used by other laboratories. Why was such a high dose used and how was the mortality rate?

-Electrode implantation: where and what was the reference?

-Survival time after KA injection was rather variable: dKA: 27 days +/- 14; vKA: 24+/- 14. Why such a big variation with respect to survival times. Since the model is progressive with time, this does not make sense.

-Tissue processing (line 238): Why were animals killed by cervical dislocation followed by transcardial perfusion?? Usually there is deep anaesthesia followed by perfusion. Why was there such a big variation in survival times +/- 150 days? Since the pathology is progressive in this animal model, it makes any comparison very difficult.

-How many tissue sections were used for quantification of Timm staining and granule cell dispersion? Please, check whether this affect statistical analysis

- Results: Line 269: “Epileptic pathology affects human patients and animal models in different dimensions, including anatomically, electrophysiologically, and cognitively.” This is a strange sentence!

- Line 275: “Aberrant sprouting of mossy fibers (mossy fiber sprouting, MFS; Figure 1a) as well as dispersal of the dentate gyrus granule cell layer (granule cell dispersion, GCD; Figure 1c) are classical histopathologies (Houser,1990; Schiebel 1974).” The main pathology of mTLE is cell death in CA1, CA3 and hilus and this is not mentioned at all, which this referee finds rather puzzling. In addition, there is widespread interneuron loss after dorsal KA injection, which was not investigated in the present manuscript.

## Author Response

To the reviewers:

Thank you for your comments on the manuscript. It has been substantially revised to address your concerns. Because of your feedback, we believe the manuscript is stronger and clearer. Specific responses to individual comments are listed below, but to summarize the major changes:

• references and discussion, especially comparing phenotypes of other epilepsy models, have been expanded

• images have been captured at high quality and their formatting changed to enhance clarity

• analysis of ictal phenotypes has been done at the seizure level

• to address concerns of disease progression, ictal data from a later timepoint is now also included

__________________

-Number of animals for each experiment should be given in the text, and not only at Fig. captions

Done.

-I found interesting how authors use power analysis in the narrative. Please, include specific details in methods

Done.

-With almost 20 seizures per day, I wonder how informative is cognitive performance?

While others have noted cognitive deficits dissociated from seizure burden, it is very possible that seizure burden is playing a major role in spatial deficits (discussed in the manuscript, beginning on line 564). It is noteworthy, however, that neither group displayed a deficit on the object recognition test and that other co-morbidities (hyperactivity and anhedonia) were differently expressed between dKA and vKA animals, despite similar seizure burdens.

-The paper is poorly referenced in general. I would discuss a bit more previous characterization of the intra-hippocampal Kainate model and possibly the i.p kainate model. For intance, our data with the ip model show specific cognitive deficits in the abscence of seizures (see Inostroza et al. PLoS ONE 2011, PLoS ONE 2012 and JN 2013). You don't need to cite our papers, I just refer for comparison with the ip model. Also, consider some of the following citations for the intra-hippocampal model: Suzuki et al., 1995 (1st description of iKA model

Riban et al., 2002 (ephys iKA model)

Heinrich et al. JN 2006, 2011(time course of dispersion)

Häussler et al CerCor 2012 (time course of LFP). We also feel it may be useful to dicuss on the intra-amygdala KA model. Consider some of these: G. Mouri, ... D Henscehll 2008(intra-amygdala KA)

G. Liu, ... J McNamara et al 2013 (used the same model, intra-amygdala KA)

Done. References have been expanded and additional comparisons to other models have been added (lines 461 - 469, 491 - 509, 586 - 590, 598 - 609).

-Fig.1: Some images are poor quality and out of focus. Poor statistical differences suggest large dispersion, please show single data points. Please, clarify how are data analyzed (section/animal).

Done. Images have been improved and how the data was analyzed has been clarified.

-Figure 1a - vKA, ventral DG - it is difficult to resolve the GC layer versus the IML in this panel. Could you add some dotted lines around the GCL to help guide the reader?

Done.

- Figure 1a: The sections of the “dorsal” DG do not represent dorsal hippocampal sections. There is granule dispersion missing in the dorsal DG/dKA section. Figure 1b. The quality of the DAPI-stained sections is rather poor.

All dorsal sections represent approximately 1.8mm posterior to bregma. Note that sections with more modest granule cell dispersion were chosen for Figure 1a, in order to more clearly illustrate MFS.

Images have been improved.

-Line 84 - Change “lead” to “led”

Done.

-Throughout consider revising the text to say “LFP monitoring” versus “depth EEG”.

Done.

-Fig.2: the n here should be seizures. A nested design (seizure/animal) may affect statistical report

Analysis at the seizure level has been added.

-Line 38: “Following a latent period, this model produces spontaneous, recurring seizures as well as classic histopathological features also observed in human TLE: sprouting of mossy fibers (mossy fiber sprouting, MFS) and dispersion of granule cells (granule cell dispersion, GCD) (Bouilleret et al., 1999)”.

Human mTLE is accompanied by hippocampal sclerosis which is characterized by selective cell death and not only by mossy fiber sprouting and granule cell dispersion, but this feature is not mentioned at all.

Sentence has been revised.

-Methods: Why were both sexes used in this study? Usually only males are used for this mTLE model. Please, clarify

We agree that in many previous studies, including some of the first descriptions of the model, only male subjects have been used. However, in multiple laboratories (Andersson et al 1991, Klein et al 2015, Li et al 2017, Bui et al 2018), either both sexes or exclusively female subjects have been included, demonstrating that the IHKA is viable in both male and female rodents. Additionally, the present work is supported by grants from the National Institutes of Health which requires “strong justification...to study only one sex” (NOT-OD-15-102, 2015). More broadly, there is presently a push to rectify the historical underrepresentation of female subjects in both animal and clinical studies, and the goal of making scientific research more inclusive must be carefully weighed against the experimental and logistical concerns of studying both sexes. We do not believe enough evidence is available to warrant the exclusion of female subjects in this study.

-Kainate (KA) injection: here 100nL of 20 mM kainate were injected. This is twice the volume and amount which is used by other laboratories. Why was such a high dose used and how was the mortality rate?

There was zero acute mortality. This has been added to the methods section (line 104).

Please note that we have found large strain differences in sensitivity to KA, and we recommend dosing KA levels to the particular strain for each study.

-Electrode implantation: where and what was the reference?

Signal was obtained from the local differential of the two wires of the twisted wire electrode. This is now included in the Methods (line 119).

-Survival time after KA injection was rather variable: dKA: 27 days +/- 14; vKA: 24+/- 14. Why such a big variation with respect to survival times. Since the model is progressive with time, this does not make sense.

To address this concern, we have analyzed data from a later timepoint as well, and continue to find no substantial difference between the models in electrographic seizures (lines 337-345). Note that the low animal numbers (and resulting low number of behavioral seizures) precluded statistical analysis of LBS at this later timepoint.

Please also note that variability in time post-KA was matched between dKA and vKA groups at the earlier timepoint as well, and therefore the statistical comparison between dKA and vKA seizures is robust.

-Tissue processing (line 238): Why were animals killed by cervical dislocation followed by transcardial perfusion?? Usually there is deep anaesthesia followed by perfusion. Why was there such a big variation in survival times +/- 150 days? Since the pathology is progressive in this animal model, it makes any comparison very difficult.

While the laboratory now utilizes deep anesthesia prior to perfusion, cervical dislocation was the only euthanasia technique compatible with perfusion available at the time of the experiments.

Anatomical pathology data was analyzed in a paired (within animal) manner, and is robust to differences in survival times.

-How many tissue sections were used for quantification of Timm staining and granule cell dispersion? Please, check whether this affect statistical analysis

Two sections per animal, now described in Methods ln 259. We utilized a paired design to compare pathology near the site of dorsal targeting vs. the site of ventral targeting within an animal. Lines connecting the data points from individual animals have been added to the graphs to aid the reader.

- Results: Line 269: “Epileptic pathology affects human patients and animal models in different dimensions, including anatomically, electrophysiologically, and cognitively.” This is a strange sentence!

Sentence has been revised.

- Line 275: “Aberrant sprouting of mossy fibers (mossy fiber sprouting, MFS; Figure 1a) as well as dispersal of the dentate gyrus granule cell layer (granule cell dispersion, GCD; Figure 1c) are classical histopathologies (Houser,1990; Schiebel 1974).” The main pathology of mTLE is cell death in CA1, CA3 and hilus and this is not mentioned at all, which this referee finds rather puzzling. In addition, there is widespread interneuron loss after dorsal KA injection, which was not investigated in the present manuscript.

Sentence has been revised.
