# Supplementary material for: Targeting the Mouse Ventral Hippocampus in the Intrahippocampal Kainic Acid Model of Temporal Lobe Epilepsy
Source: eNeuro. 2018 Aug 8;5(4):ENEURO.0158-18.2018. doi: 10.1523/ENEURO.0158-18.2018 (PMC6102375; doi:10.1523/ENEURO.0158-18.2018)
Supplement: Extended Data 1 — Automated ictal spike counter. We have provided the code (created in Matlab 2014b on a Windows 7 computer) to run our custom automated spike counter, used in this article to quantify electrographic seizures. The code runs as a Matlab script, which launches a customizable user interface. A detailed user guide accompanies the code. A copy of the code and user guide can also be found at the URL listed in the Methods – Code Accessibility section. Download Extended Data 1, ZIP file. [file sup_enu-eN-NWR-0158-18-s02.zip › Electrographic Seizure Analyzer/Blinded - Electrographic Seizure Analyzer.pdf]

Electrographic Seizure Analyzer

User Guide

Custom Electrographic Seizure Analyzer

The software described here was created in Matlab 2014b, and does not require the installation of any Toolboxes. It does use the function peakseek.m, which can be found at the Mathworks File Exchange at:
https://www.mathworks.com/matlabcentral/fileexchange/26581-peakseek?focused=5176885&tab=function

The software allows for flexibility in file analysis through a large number of parameter settings. The most commonly used settings are coded as default settings. However, this is no guarantee that these settings will lead to a meaningful analysis for every data set, and it is up to the user to ensure that the analysis performed by the software is meaningful and accurate.

For any questions regarding the software, please contact [redacted for double blind review].

Table Of Contents

Custom Electrographic Seizure Analyzer

Table Of Contents

Run the GUI: findallofthem.fig

- An Overview
- Step 0: Settings
- Step 1: Choose Files
- Step 2: Determine Cutoffs
- Step 3: Run Analysis

Analysis & Statistics

Matlab files

Run the GUI: findallofthem.fig

An Overview

The user interface and underlying code can be used to load in a directory containing multiple files of data, and analyze the data to detect temporal events (‘positive events’ are detected separately from ‘negative events’) as well as noise (e.g. movement artifact). Positive and negative events are then combined into a set of ‘clean events’, excluding the noise.

This process can be done automatically for all files in a given directory, and the analysis of each file, including varying descriptive statistics on event occurrence and duration are stored in a .mat file, as well as .csv file in a user specified location.

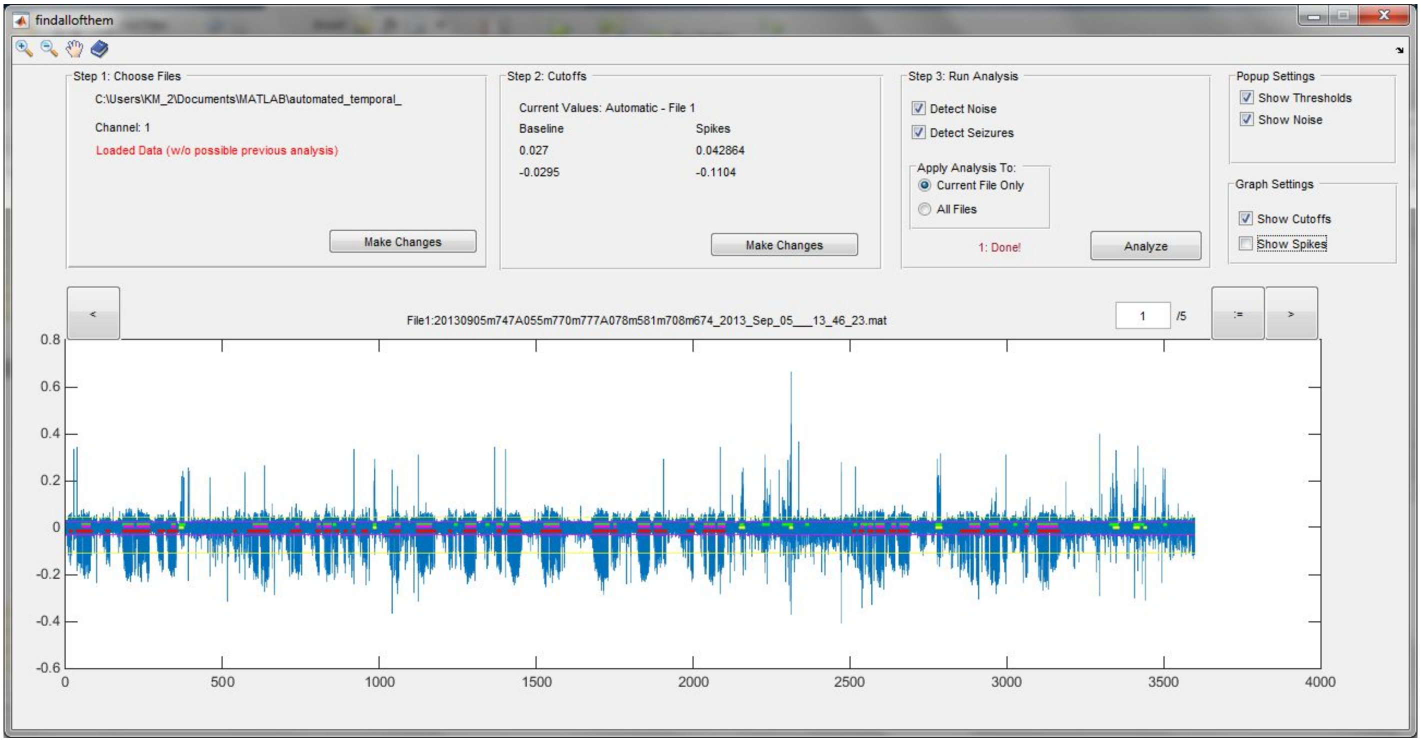

Always follow the following steps when using the program:

Step 0: Change settings by clicking the book icon. (This is also where you can specify the output folder.)

Step 1: Choose the files to load in (with or without any prior analysis done).
Use the arrows ‘<’ and ‘>’ to flip through the different files .

Step 2: Automatically analyze the data to determine the spike cutoffs, baseline cutoffs, as well as other thresholds used in noise detection.
It is recommended to verify that the automatic thresholds calculated are a good fit for the other files in the folder (common) by using the arrows ‘<’ and ‘>’ to see them overlayed on these files.

Step 3: Use the pre-analysis in step 2, as well as the settings specified in step 0, and analyze either the current file displayed, or all files in the folder. All analysis and any statistics on the analysis is stored in a .mat file in the specified output folder. The descriptive statistical analysis is also exported in .csv and .xls format.

- You can use the arrows ‘<’ and ‘>’ to see the various files in the folder.
- You can use the menu bar’s *magnifying glass* and *hand* icon to zoom in/out or move the graph.

Step 0: Settings

Access the settings menu by clicking on the book icon in the menu bar.
Settings are separated by the part of the program they are used in.

- The values displayed are loaded from *"DefaultSettings.m"* and if you consistently want different values, you can edit them directly in this file to change the default settings.
- In case of a previously loaded analysis, the displayed settings are those saved from that session.
- If you change any value in the middle of analyzing a set of files, the program will "reset" in order to ensure that the analysis of all files within the same folder is done with consistent settings.
- If you wish to store analysis of the same files with different parameters, simply change the output folder prior to starting the analysis.
- The default settings values are used in the descriptions below (in blue) to more easily communicate what each settings does. These are initial settings only, and should be adjusted by the user as appropriate.

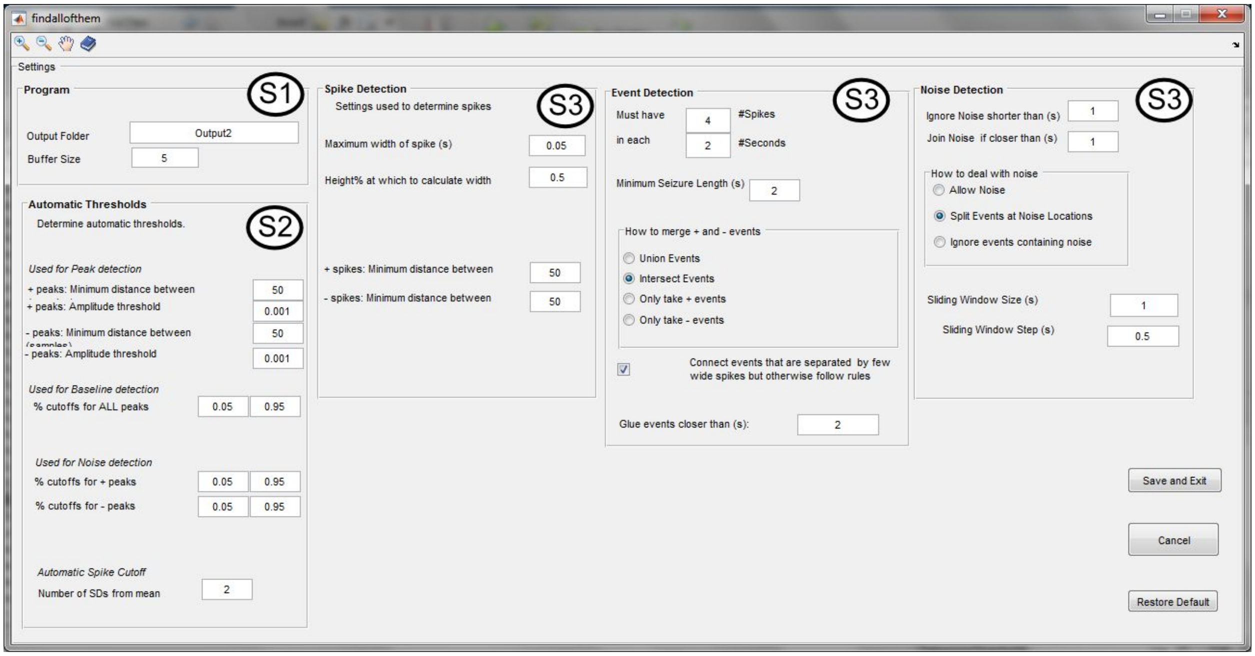

S1 - Load in Files

Label	Description
'Output Folder'	subfolder created within the input folder and used for storing the analysis and accompanying session settings
'Buffer Size'	The number of files that will be kept in memory for a given folder.<br>E.g. if you have a folder with 100 files, but set your buffer to 5, then only the 5 most recently viewed files in the folder are kept in memory, so you can quickly switch between them. When you move to a file not in the buffer, the "oldest" buffer item is removed from the buffer, and the new file is placed in its place.

S2 - Automatic Cutoffs

Please note that the default values are used in the descriptions (in blue) to more clearly communicate what the setting does. However, it is up to the user to adjust these settings as appropriate.

Label	Initial Default Setting	Description
**Used for peak detection**		
'Minimum distance between peaks'	50	Measured in #samples. Helps smooth the data
'Amplitude threshold'	0.001	Set low, since we initially want to detect most data points<br>*Both are settings used in peakseek.m*
**Used for baseline detection**		
'%cutoff for ALL peaks'	0.05<br>0.95	The baseline is made up of all data values between 5% of the data and 95% of the data in a cumulative histogram.
**Used for noise detection**		
'%cutoff for + peaks'	0.05<br>0.95	Find cutoffs such that 5%-95% of all positive peaks in a cumulative histogram fall within the range.
'%cutoff for - peaks'	0.05<br>0.95	Find cutoffs such that 5%-95% of all negative peaks in a cumulative histogram fall within the range.<br>*Both cutoffs are used for identifying noise in the signal.*
**Automatic Spike cutoff**		
'Number of SD's from mean'	2	Positive/negative peaks are considered spikes if they exceed 2SD from the positive/negative mean respectively.

S3 - Spike Detection

Label	Initial Default Setting	Description
'Maximum width of spike (s)'<br>'height% at which to calculate width'	0.05<br>0.5	A peak's width cannot exceed 0.05s measured at 50% of its maximum amplitude to be counted as a spike.
'+ Maximum distance between'<br>'- Maximum distance between'	50<br>50	Spikes whose peaks are within 50 samples are considered part of the same spike.<br>*Note that separate values can be used for positive and negative spikes*

S3 - Event Detection

Label	Initial Default Setting	Description
'Must have _4_ spikes'<br>'In each _2_ seconds'		An event is a series of rapid spikes, such that in each 2s sliding window of the event there are at least 4 spikes.<br>Note: + events and - events are considered separately and merged later.
'Minimum seizure length (s)'	2	An event is only included if it lasts at least 2s.
'How to merge + and - events'		Given a list of +events and -events we create a list of 'events' by either:
'Union events'		➢ Only overlap of +events and -events counts
'Merge events'		➢ Anytime at least +event OR -event counts
'Only take + events'		➢ Ignore the -events
'Only take - events'		➢ Ignore the +events

Label	Initial Default Setting	Description
'Connect events ... few "wide spikes"		Allows us to connect events that together would have satisfied the sliding window criteria IF we loosened the spike criteria to allow for wider spikes
'Glue events closer than (s)'	2	Connect events that have a gap less than 2s in-between

S3 - Noise Detection

'Ignore noise shorter than (s)'	1	Don't alter events if short noise less than 1s in duration occurs simultaneously.
'Join noise if closer than (s)'	1	Connect two periods of noise if they have a gap less than 1s between them.
'How to deal with noise'		Once we have a set of events, and a set of noise occurrences that may or may not overlap with these events, then we can create a set of 'clean events' by:
'Allow noise'		➢ Ignore the noise, leaving events unchanged
'Split events at noise locations'		➢ Events are trimmed to make sure there is no noise simultaneous to any of the events
'Ignore events containing noise'		➢ Only take those events that don't overlap with noise
'Sliding window size'<br>'Sliding window step'	1<br>0.5	In order to detect noise, use a 1s sliding window and go through all the data, moving this window 0.5s in each step. For each sliding window a set of metrics on the data in the window is calculated. These metrics are used to determine if *noise* occurred in the window. Decreasing the step size will lead to increased resolution of noise location at the cost of more computing time and required memory size.

Please note that the *noise detection* is hardcoded in the file FindNoise.m
You can change which metrics to use to define noise in the code of this file and use the popup noise window to determine its effectiveness.

Buttons

'Save and Exit'	Stores the new settings and closes the settings window.
'Cancel'	Closes the settings window without saving any potential changes you made.
'Restore Default'	Load in default settings from 'DefaultSettings.m'<br>*Note: This implicitly saves the default settings and cannot be undone by 'cancel'*

Step 1: Choose Files

Select the .mat files we want to analyze, and if desired, load in any prior analysis for the files.

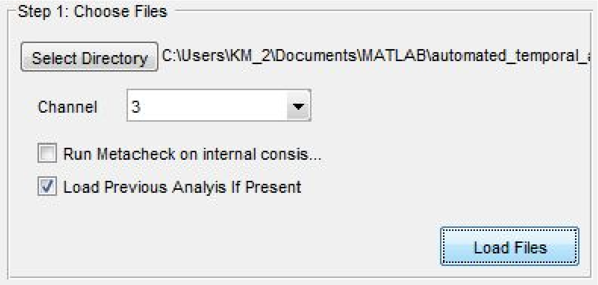

- Select a directory that contains the .mat files of interest
  - All subdirectories in the selected directory are ignored
  - .mat files are expected to contain the following components:

'number_of_channels'	number of separate channels with data
'fs'	fs is the number of samples per second
'sbuf'	sbuf(i,j) is datapoint i for channel j
'trdata'	trdata(i).timestamp is [file start time, …, file end time] *Only first and last entry are extracted. The values are used to calculate the file duration needed for various statistics on the file.*
'ledon', 'ledoff', 'ledactive'	ledon(i) are stimulation parameters for channel i *These are not used in analysis, but rather used to verify that data files are internally consistent and should be analyzed together.*

- Select the recording "channel" within these .mat files you are interested in

- "**Run metacheck**" will result in a preanalysis of the files, to ensure that ALL files for the selected channel match with respect to stimulation parameters and sampling rate.
  NB: Unchecking will remove the need for parameters 'ledon','ledoff','ledactive' to be present

- "**Load previous analysis if present**" will allow you to load in (partial or complete) analysis done and saved for this directory & channel during a previous session. The program attempts to load in the analysis as well as all settings used during the analysis from the specified output directory. If none exists, this will be reported and the files are loaded without analysis and with default settings.

- "**Load Files**" results in the program displaying the first file in the directory, as well as the file count.
  If previous analysis was loaded in, then events and noise occurrences are displayed in the graph, and settings values prepopulate the cutoff values in step 2.

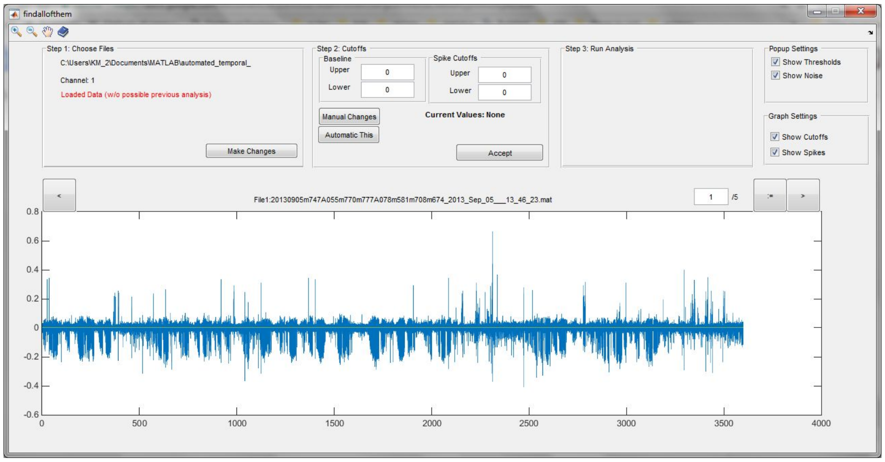

Step 2: Determine Cutoffs

The software will analyze the currently displayed trace in order to automatically find a set of threshold values to specify what counts as baseline and what counts as spikes (both positive and negative). The analysis can be displayed on the screen in a popup window, and the automatically generated values can be changed manually. When determining these threshold values, it is important to select a trace that is reflective of the files to be analyzed.

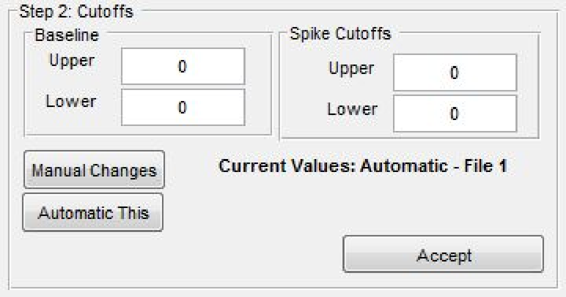

- "**Automatic This**" will analyze the recentered data.
  The analysis will be displayed in a popup if "**Show Thresholds**" is checked in the upper right side, under 'Popup Settings'.

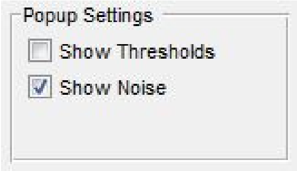

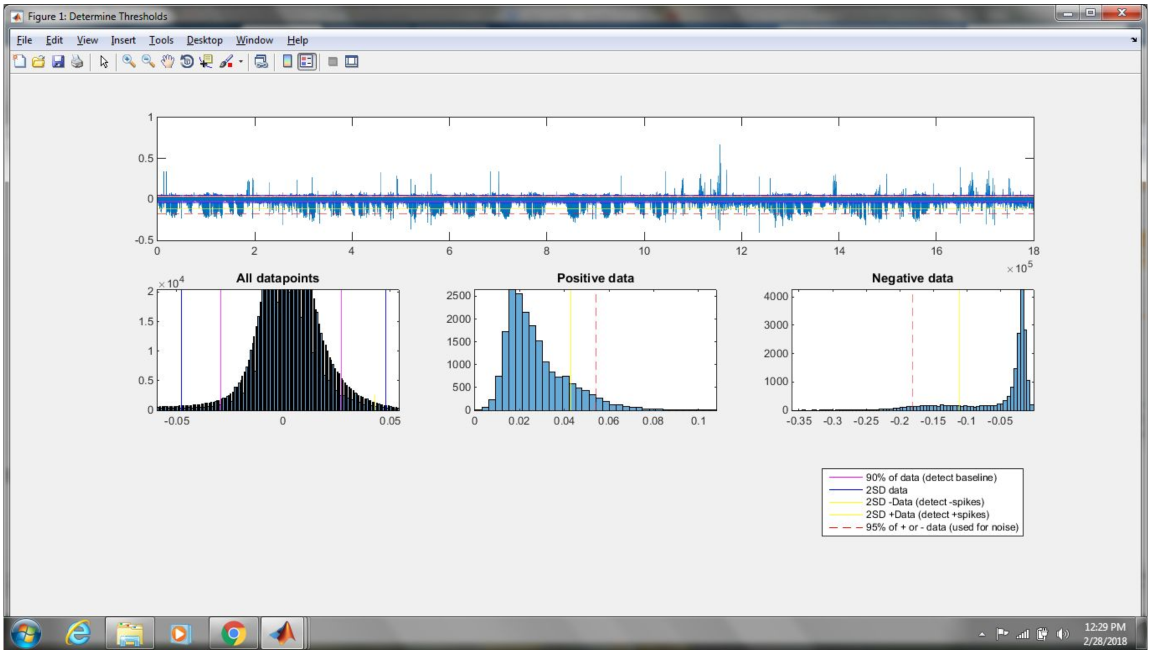

- 90% of data — captures the baseline of the data
- 2SD data — not currently used
- 2SD +data — any +peak exceeding this threshold is picked up as a **+spike**
- 2SD -data — any -peak exceeding this threshold is picked up as a **-spike**
- 95% of + or - data — creates automatic thresholds used in noise detection

Once the thresholds are determined, they will populate the textboxes as below:

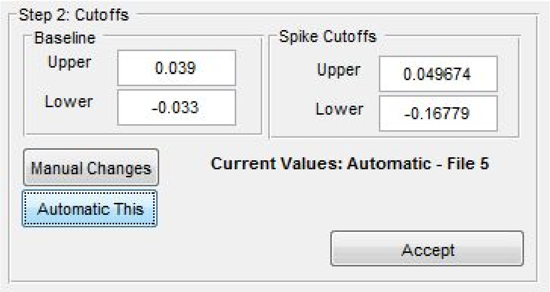

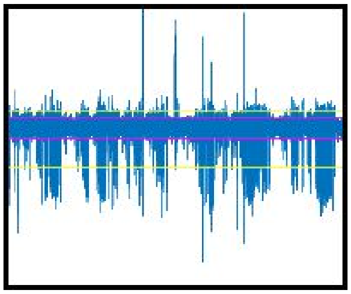

- Adjust the values manually as desired. You can view the traces of the current cutoffs in the main display (baseline cutoffs are shown in magenta, and spike cutoffs are shown in yellow)
- Once satisfied, hit "**Accept"**
- The software will display the source of the thresholds (e.g. "Automatic - File 5" vs "Manual") and will store this within the analysis files. If you change these threshold values after starting the analysis, it will RESET any completed analysis to ensure consistent settings throughout the analysis. To avoid this, choose a different 'output directory' prior to changing any threshold values.

Step 3: Run Analysis

Automatically analyze either the current file, or all files in the directory to find all events and all noise occurrences. Events are identified by searching for prolonged sections of sufficiently dense spikes, as set in steps 1 & 2.

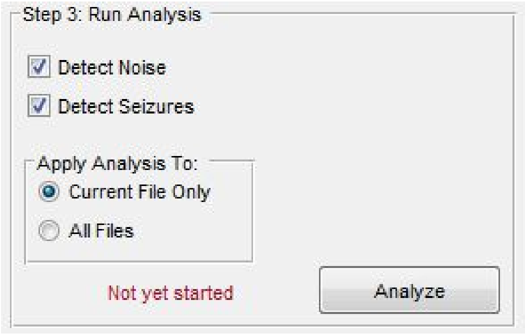

- For full analysis, keep both **'detect noise'** and **'detect seizures'** checked
- As part of the noise detection, a popup window will be shown if **'Show Noise'** is checked in the upper right side, under 'Popup Settings'.

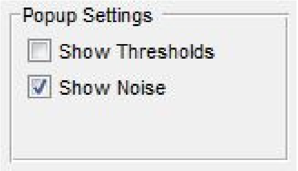

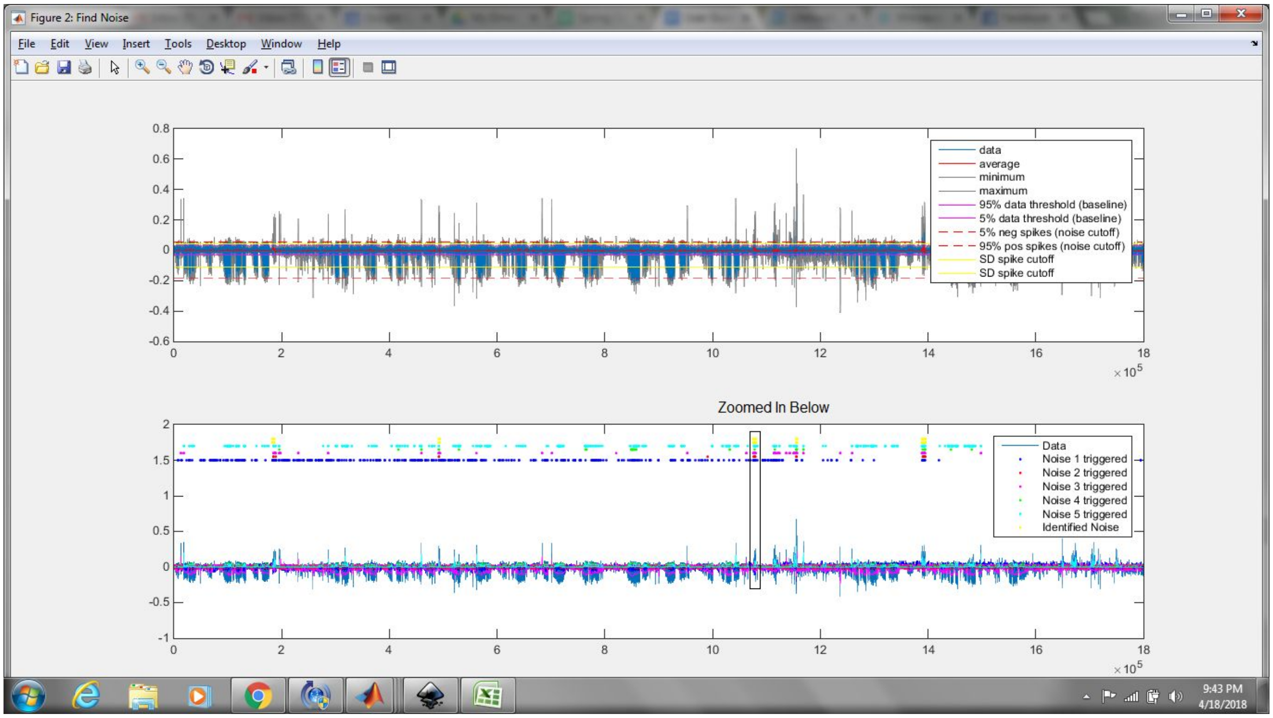

The top figure displays the trace with the cutoffs and some general metrics calculated on the data points in a sliding window. You can make adjustments to the graph in the file *'FindNoise.m'.*

The bottom figure displays the identified noise based on a variety of triggers.
The image to the right zooms in on a noisy section of the trace.
Note that each trigger metric is displayed in a different color (blue, red, magenta, green, cyan). The dots (in matching colors) indicate whether *that* metric triggered noise detection. The yellow dots indicate that we conclude this part of the trace to be in a noise state, and the yellow line indicates the identified noise segment, specifying the start and end. Noise triggers are hardcoded in the file *'FindNoise.m'*, and the currently used ones are further explained below.

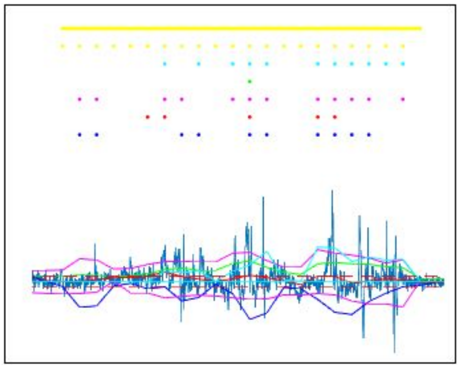

Currently, the combined noise (yellow) is a combination of trigger 2 (average window value exceeds a set threshold) and 3 (average positive value *or* average negative value exceeds threshold). Once noise is triggered, the *number of direction changes (trigger 1)* is used to find the beginning and end of the noise occurrence.
Note that many other metrics are calculated in the file 'FindNoise.m' for the trace, but based on our observations we picked these simple metrics to identify noise for our signals.

- Analysis is displayed, where all events and noise occurrences are overlaid with colored lines

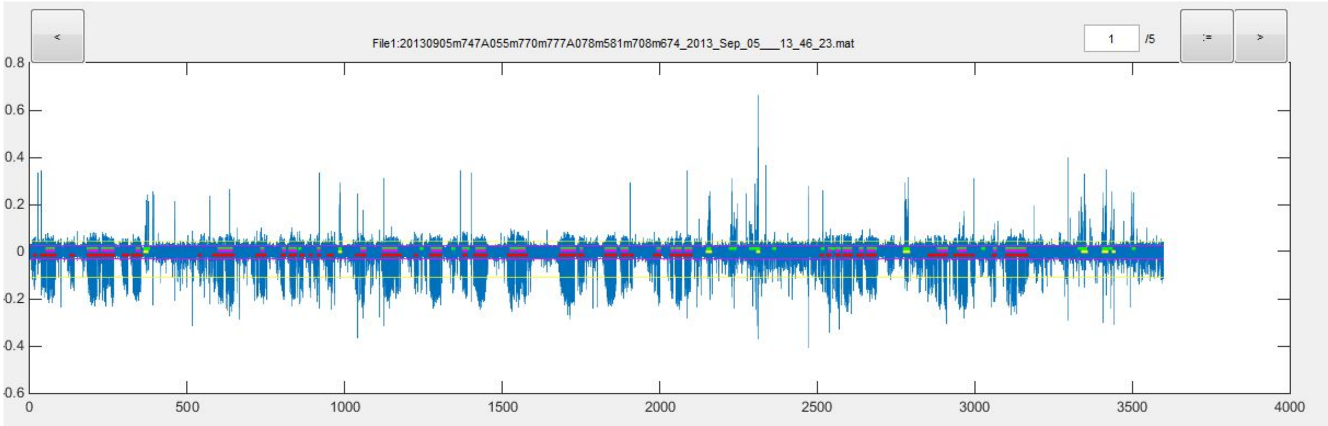

Thick Yellow ➢ noise occurrences
Thick Red ➢ negative events
Thick Green ➢ positive events
Thick Magenta ➢ combined events (obtained from 'positive events' and 'negative events' by either union, intersection, or purely positive or negative as specified in Settings.*)*

These are reduced by any noise as specified in Settings.

*Note that Red and Green lines are USED to determine the Magenta lines, which are **the events** of interest.*

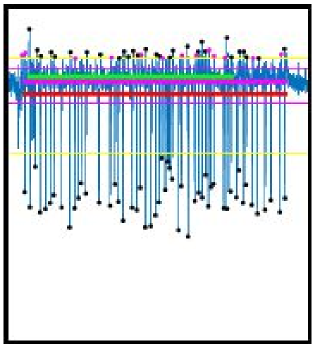

The image to the left shows a positive event (green), a negative event (red) and the resulting combined event (magenta) which was obtained with the setting "Only take negative events". (Note that the thin yellow line is the spike threshold value.)

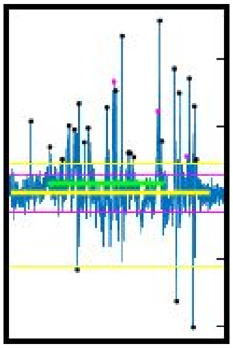

The image to the left shows a noise occurrence in the signal. Even though positive spikes triggered a potential positive event (thick green line), there is no resulting combined event (i.e., no thick magenta line). Rather, the entire noise is flagged by the thick yellow line. (Note that the thin yellow line is the spike threshold value.)

- Use the buttons below to zoom in, zoom out, or pan the graph

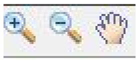

- 'Show cutoffs' Toggle display of the cutoffs
  Magenta lines ➢ baseline
  Yellow lines ➢ spike cutoff as determined in Settings

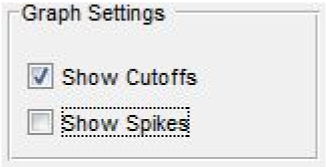

- ‘Show spikes’ Toggle display of all spikes
  - black dots ➢ spikes that meet width criteria
  - pink dots ➢ peaks that are too wide to count as spikes

- All calculated metrics on individual events and noise occurrences, as well as statistics on the file as a whole are stored in the ‘output’ directory using the file format ‘Analysis_ch1.mat’ and ‘Analysis_ch1.csv’ and ‘Analysis_ch1.xls’

- When additional files in the directory are analyzed, their metrics are appended in these same files.
  Note that if settings change, the files will be overwritten instead. To avoid this you can change the ‘output directory’ prior to any settings changes.

Analysis & Statistics

The following information and descriptive statistics are calculated and stored for each event (in .xls format, in .csv format, as well as within the .mat file in the variable info.fileinfo.EventStats). The .xls format includes column headers.

Start date	Start date of event
Start time	Start time of event
End date	End date of event
End time	End time of event
Duration	Duration of event (in s)
#+Peaks	Number of positive peaks in the event
#-Peaks	Number of negative peaks in the event
#+wide peaks	Number of positive peaks, exceeding width threshold, in the event
#-wide peaks	Number of negative peaks, exceeding width threshold, in the event
#+spikes (=#+narrow peaks)	Number of **positive spikes** (peaks that are narrow enough) in the event
#-spikes(=#-narrow peaks)	Number of **negative spikes** (peaks that are narrow enough) in the event
average width +wide peaks	Average width (s) of the positive wide peaks in the event
average width -wide peaks	Average width (s) of the negative wide peaks in the event
%+spikes (wrt +peaks in event)	Percentage of all positive peaks in the event that were identified as spikes
%-spikes (wrt -peaks in event)	Percentage of all negative peaks in the event that were identified as spikes
+spikes/s	Positive spike rate in the event
-spikes/s	Negative spike rate in the event
+peaks/s	Positive peak rate in the event (includes both spikes as well as wide peaks)
-peaks/s	Negative peak rate in the event (includes both spikes as well as wide peaks)

The following statistics are calculated for the file as a whole and stored in .mat file in Info.FileInfo.

TimeInEvent (s)	Total duration of all events in the file (s)
PercInEvent (%)	Percentage of the file spent in events (%)
TimeInNoise (s)	Total duration of all noise in the file (S)
totalposspikes	Total number of positive spikes in the file (not only those in events, but ALL)
totalnegspikes	Total number of negative spikes in the file (not only those in events, but ALL)
posspikerate	Number of positive spikes / s for the entire file
negspikerate	Number of negative spikes / s for the entire file

Matlab files

The code requires several matlab files to run. There are comments prior to each function explaining the use, inputs, and outputs of the function, as well as in-line comments explaining the code further.

The following is a list of all files in order of appearance.

- findallofthem.fig — User Interface for Automated Temporal Analysis
  Open with GUIDE to change user interface

- findallofthem.m — Main code, which interacts with the User Interface and calls all other functions

- DefaultSettings.m — Load in the default settings preset in this file to be used in the analysis.
  You can change preset values once through the GUI Settings Menu, or change them directly in this file for a more permanent change.

- AnalyzeDirectory.m — Analyze given input directory and return a list of all .mat files, ignoring all subdirectories. If specified, check that files share the same recording parameters (ledon, ledoff, ledactive, fs)

- SpikeFinder.m — Find the location and width of ALL peaks in the data that adhere to the spikes settings. *(Uses peakseek.m)*

- DetermineThresholds.m — Use currently loaded data-file to calculate automatic cutoffs for baseline, and spike-identification
  (Uses peakseek.m)

- FindEvents.m — Find positive events and negative events, based on event settings and the positive spikes and negative spikes respectively

- FindNoise.m — Find noise occurrences based on noise identification settings as well as various metrics hardcoded in this file. Please edit this file directly to fine tune any noise detection for your data set.

- MergeEvents.m — Merge the positive and negative events according to the settings, and cross-reference the results with noise occurrences to return a cleaned event list

- peakseek.m (obtained from the Mathworks File Exchange at https://www.mathworks.com/matlabcentral/fileexchange/26581-peakseek?focused=5176885&tab=function)
